# Supplementary material for: Polyketide synthase gene clusters in the ericoid mycorrhizal fungus Oidiodendron maius: genome-wide prediction, expression profiling, and functional characterization of knockout mutants
Source: Front Plant Sci. 2026 Sep 16;17:1924430. doi: 10.3389/fpls.2026.1924430 (PMC13623903; doi:10.3389/fpls.2026.1924430)
Supplement: Supplementary file 1 [file SupplementaryFile1.zip › Supplementary Materials/Supplementary Folder 1.zip!/Oidma1_AssemblyScaffolds_Repeatmasked/knownclusterblast/cluster41/jgi.p_Oidma1_44993_mibig_hits.html]

| MIBiG Protein | Description | MIBiG Cluster | MiBiG Product | % ID | % Coverage | BLAST Score | E-value |
| --- | --- | --- | --- | --- | --- | --- | --- |
| EHA19289.1 | hypothetical\_protein | BGC0001124 | Pyranonigrin\_E\_biosynthetic\_gene\_cluster | 61 | 83.2034018427 | 2809 | 0.0 |
| AKL78824.1 | GLPKS3 | BGC0001187 | Xenolozoyenone\_biosynthetic\_gene\_cluster | 50 | 100.566973777 | 2737 | 0.0 |
| EWG54266.1 | hypothetical\_protein | BGC0001190 | Fusaric\_acid\_biosynthetic\_gene\_cluster | 34 | 85.5067328136 | 1218 | 0.0 |
| ACD39774.1 | reducing\_polyketide\_synthase | BGC0000134 | Radicicol\_biosynthetic\_gene\_cluster | 33 | 86.0028348689 | 1095 | 0.0 |
| AIA58899.1 | HRPKS | BGC0001141 | Brefeldin\_biosynthetic\_gene\_cluster | 32 | 86.6052445074 | 1024 | 2.5e-298 |
| CBX87032.1 | BcBOA9\_protein | BGC0001243\_c3 | Botryenalol\_/\_botrydial\_/\_dihydrobotrydial\_/\_botryendial\_/ | 31 | 85.8610914245 | 1002 | 1.3e-291 |
| CAP95405.1 |  | BGC0001404 | Sorbicillin\_biosynthetic\_gene\_cluster | 38 | 46.4564138909 | 784 | 4.4e-226 |
| AAB08104.3 | polyketide\_synthase | BGC0000155\_c3 | T-toxin\_biosynthetic\_gene\_cluster | 35 | 53.6498936924 | 764 | 4.7e-220 |
| BAC20566.1 | polyketide\_synthase | BGC0000039 | Compactin\_biosynthetic\_gene\_cluster | 38 | 46.2083628632 | 758 | 2e-218 |
| ACS68554.1 | hybrid\_PKS-NRPS\_protein | BGC0001026 | NG-391\_biosynthetic\_gene\_cluster | 38 | 45.4642097803 | 748 | 2e-215 |
| BAV32159.1 | polyketide\_synthase | BGC0001373 | Sordarin\_biosynthetic\_gene\_cluster | 37 | 47.2360028349 | 745 | 3e-214 |
| AGC95324.1 | CurS1 | BGC0000045 | Dehydrocurvularin\_biosynthetic\_gene\_cluster | 37 | 47.4486180014 | 742 | 2.5e-213 |
| ACD39758.1 | reducing\_polyketide\_synthase | BGC0000076 | Hypothemycin\_biosynthetic\_gene\_cluster | 36 | 47.3423104181 | 737 | 4.7e-212 |
| ACD39767.1 | reducing\_polyketide\_synthase | BGC0000077 | Hypothemycin\_biosynthetic\_gene\_cluster | 36 | 47.3423104181 | 737 | 6.2e-212 |
| EHA28244.1 | hypothetical\_protein | BGC0001143 | Azanigerone\_biosynthetic\_gene\_cluster | 36 | 45.8894401134 | 735 | 1.8e-211 |
| ABA02239.1 | polyketide\_synthase | BGC0000098 | Monacolin\_K\_biosynthetic\_gene\_cluster | 37 | 45.4996456414 | 734 | 5.2e-211 |
| AAD34559.1 | polyketide\_synthase | BGC0000089 | Lovastatin\_biosynthetic\_gene\_cluster | 38 | 45.1452870305 | 733 | 8.9e-211 |
| AKC54422.1 | fumosorinone\_biosynthesis\_polyketide\_synthase | BGC0001218 | Fumosorinone\_biosynthetic\_gene\_cluster | 36 | 46.7753366407 | 733 | 8.9e-211 |
| CAL69597.1 | nonribosomal\_peptide\_synthase | BGC0001049 | Tenellin\_biosynthetic\_gene\_cluster | 36 | 47.3423104181 | 732 | 1.5e-210 |
| ABA02240.1 | polyketide\_synthase | BGC0000098 | Monacolin\_K\_biosynthetic\_gene\_cluster | 36 | 46.9525159461 | 731 | 3.4e-210 |
| AGO86662.1 | equisetin\_synthetase | BGC0001255 | Equisetin\_biosynthetic\_gene\_cluster | 36 | 45.6059532247 | 730 | 5.8e-210 |
| CBF87072.1 | polyketide\_synthase,\_putative\_(Eurofung) | BGC0001290 | Emericellamide\_A\_/\_emericellamide\_B\_biosynthetic\_gene\_cluster | 37 | 46.6690290574 | 730 | 7.5e-210 |
| AFP73394.1 | FusA | BGC0001268 | Fusarin\_biosynthetic\_gene\_cluster | 36 | 50.2126151665 | 728 | 2.2e-209 |
| AAT28740.1 | FUSS | BGC0000064 | Fusarin\_biosynthetic\_gene\_cluster | 36 | 49.8936924167 | 728 | 2.9e-209 |
| EAA65604.1 | hypothetical\_protein | BGC0000022 | Asperfuranone\_biosynthetic\_gene\_cluster | 34 | 48.440822112 | 722 | 2.7e-207 |
| ADN43685.1 | DmbS | BGC0001136 | Desmethylbassianin\_biosynthetic\_gene\_cluster | 36 | 46.8462083629 | 720 | 6e-207 |
| AHV78245.1 | LasS1 | BGC0001245 | Lasiodiplodin\_biosynthetic\_gene\_cluster | 36 | 48.7951807229 | 720 | 7.8e-207 |
| CBF80487.1 | hybrid\_PKS-NRPS\_(Eurofung) | BGC0000959 | Aspyridone\_biosynthetic\_gene\_cluster | 34 | 51.4174344437 | 717 | 6.6e-206 |
| AHV78252.1 | ResS1 | BGC0001246 | Trans-resorcylide\_biosynthetic\_gene\_cluster | 36 | 47.8738483345 | 714 | 4.3e-205 |
| BAD83684.1 | PKSN\_polyketide\_synthase\_for\_alternapyrone\_biosynthesis | BGC0000012 | Alternapyrone\_biosynthetic\_gene\_cluster | 36 | 48.3699503898 | 713 | 7.3e-205 |
| ACZ57548.1 | polyketide\_synthase | BGC0000046 | Depudecin\_biosynthetic\_gene\_cluster | 37 | 42.4875974486 | 710 | 6.2e-204 |
| CAO91861.1 | putative\_PKS-NRPS\_protein | BGC0000968 | Chaetoglobosin\_biosynthetic\_gene\_cluster | 37 | 45.7122608079 | 705 | 3.4e-202 |
| BAJ14522.1 | polyketide\_synthase | BGC0001253\_c4 | ACT-Toxin\_I\_biosynthetic\_gene\_cluster | 35 | 47.4840538625 | 703 | 9.9e-202 |
| EAW09117.1 | hybrid\_NRPS/PKS\_enzyme,\_putative | BGC0000983 | Cytochalasin\_biosynthetic\_gene\_cluster | 36 | 45.8894401134 | 703 | 9.9e-202 |
| BAC20564.1 | polyketide\_synthase | BGC0000039 | Compactin\_biosynthetic\_gene\_cluster | 36 | 43.019135365 | 701 | 3.8e-201 |
| AAV66110.2 | fusaridione\_A\_synthetase | BGC0000992 | Fusaridione\_A\_biosynthetic\_gene\_cluster | 37 | 44.8972360028 | 699 | 1.4e-200 |
| ABB90283.1 | polyketide\_synthase | BGC0001057 | Zearalenone\_biosynthetic\_gene\_cluster | 34 | 47.3068745571 | 694 | 4.6e-199 |
| EAU38971.1 | hypothetical\_protein | BGC0001122 | Isoflavipucine\_biosynthetic\_gene\_cluster | 40 | 37.4911410347 | 691 | 5.1e-198 |
| ctg1\_orf0002 |  | BGC0001068 | Pyripyropene\_A\_biosynthetic\_gene\_cluster | 39 | 37.3493975904 | 690 | 8.7e-198 |
| BAQ25466.1 | polyketide\_synthase | BGC0001280 | Betaenone\_C\_/\_betaenone\_A\_biosynthetic\_gene\_cluster | 39 | 37.1367824238 | 678 | 3.4e-194 |
| KGO40478.1 | Acyl\_transferase/acyl\_hydrolase/lysophospholipase | BGC0001205 | Communesin\_biosynthetic\_gene\_cluster | 36 | 43.0900070872 | 675 | 3.8e-193 |
| CCT72377.1 | probable\_polyketide\_synthase | BGC0001305 | Fujikurins\_biosynthetic\_gene\_cluster | 36 | 43.9404677534 | 668 | 2.7e-191 |
| EAL89230.2 | LovB-like\_polyketide\_synthase,\_putative | BGC0000129 | Pyripyropene\_biosynthetic\_gene\_cluster | 38 | 37.207654146 | 656 | 1.8e-187 |
| EAL85113.2 | hybrid\_PKS-NRPS\_enzyme,\_putative | BGC0001037 | Pseurotin\_A\_biosynthetic\_gene\_cluster | 35 | 43.6569808646 | 654 | 6.9e-187 |
| EAL85129.1 | polyketide\_synthase,\_putative | BGC0001067 | Fumagillin\_biosynthetic\_gene\_cluster | 33 | 49.8582565556 | 641 | 4.6e-183 |
| EAQ86385.1 | hypothetical\_protein | BGC0001405 | Chaetoviridin\_/\_chaetomugilin\_biosynthetic\_gene\_cluster | 38 | 34.124734231 | 637 | 8.7e-182 |
| BAK26562.1 | PKS-NRPS\_hybrid | BGC0000977 | Cyclopiazonic\_acid\_biosynthetic\_gene\_cluster | 39 | 37.1722182849 | 631 | 3.7e-180 |
| AMY15068.1 | hexaketide\_synthase\_MF-SQHKS | BGC0001339 | Squalestatin\_S1\_biosynthetic\_gene\_cluster | 34 | 43.408929837 | 620 | 1.1e-176 |
| BAD97694.1 | Aft9-1 | BGC0000003 | AF-toxin\_biosynthetic\_gene\_cluster | 36 | 42.3812898653 | 618 | 3.2e-176 |
| AAK57188.1 | MxaD | BGC0001022 | Myxalamid\_biosynthetic\_gene\_cluster | 35 | 42.5584691708 | 615 | 2.7e-175 |
| AGC45624.1 | polyketide\_synthase | BGC0001394 | Phenalamide\_biosynthetic\_gene\_cluster | 34 | 45.4287739192 | 614 | 6e-175 |
| ACR33078.1 | polyketide\_synthase | BGC0000017 | Anatoxin\_biosynthetic\_gene\_cluster | 34 | 41.9206236712 | 611 | 6.7e-174 |
| ADB12491.1 | EpoD | BGC0000990 | Epothilone\_biosynthetic\_gene\_cluster | 35 | 41.8143160879 | 605 | 3.7e-172 |
| AEE88280.1 | CurJ | BGC0000976 | Curacin\_biosynthetic\_gene\_cluster | 33 | 42.4875974486 | 604 | 8.2e-172 |
| AAT70105.1 | CurJ | BGC0001165 | Curacin\_biosynthetic\_gene\_cluster | 33 | 42.4875974486 | 604 | 8.2e-172 |
| AAF62883.1 | epoD | BGC0000991 | Epothilone\_biosynthetic\_gene\_cluster | 34 | 41.7788802268 | 603 | 1.4e-171 |
| CAD19091.1 | StiG\_protein | BGC0000153 | Stigmatellin\_biosynthetic\_gene\_cluster | 34 | 41.7788802268 | 602 | 3.1e-171 |
| AIW82279.1 | PuwB | BGC0001125 | Puwainaphycins\_biosynthetic\_gene\_cluster | 34 | 41.9560595322 | 597 | 7.6e-170 |
| ABL86391.1 | hybrid\_polyketide\_synthase\_and\_nonribosomal\_peptide\_synthetase | BGC0000999 | Heat-stable\_antifungal\_factor\_biosynthetic\_gene\_cluster | 34 | 41.8143160879 | 596 | 2.2e-169 |
| AGC45619.1 | polyketide\_synthase | BGC0001394 | Phenalamide\_biosynthetic\_gene\_cluster | 33 | 43.1254429483 | 592 | 2.5e-168 |
| AGC45621.1 | polyketide\_synthase | BGC0001394 | Phenalamide\_biosynthetic\_gene\_cluster | 34 | 42.3458540043 | 592 | 2.5e-168 |
| ADB12492.1 | EpoE | BGC0000990 | Epothilone\_biosynthetic\_gene\_cluster | 34 | 42.629340893 | 592 | 3.2e-168 |
| AAF62884.1 | EpoE | BGC0000991 | Epothilone\_biosynthetic\_gene\_cluster | 34 | 42.629340893 | 591 | 4.2e-168 |
| ABM21570.1 | CrpB | BGC0000975 | Cryptophycin\_biosynthetic\_gene\_cluster | 33 | 43.7632884479 | 589 | 1.6e-167 |
| AAF26922.1 | polyketide\_synthase | BGC0000988 | Epothilone\_biosynthetic\_gene\_cluster | 34 | 42.629340893 | 589 | 1.6e-167 |
| AAF26921.1 | polyketide\_synthase | BGC0000988 | Epothilone\_biosynthetic\_gene\_cluster | 35 | 36.9241672573 | 585 | 3e-166 |
| ACB46196.1 | polyketide\_synthase | BGC0000989 | Epothilone\_biosynthetic\_gene\_cluster | 33 | 42.629340893 | 584 | 8.7e-166 |
| AIT55263.1 | GulF | BGC0000072 | Gulmirecin\_biosynthetic\_gene\_cluster | 33 | 45.3933380581 | 583 | 1.5e-165 |
| ACB46195.1 | polyketide\_synthase | BGC0000989 | Epothilone\_biosynthetic\_gene\_cluster | 35 | 36.8887313962 | 582 | 2.5e-165 |
| CCE88376.1 | polyketide\_synthase | BGC0001034 | Pellasoren\_biosynthetic\_gene\_cluster | 32 | 46.0666194189 | 579 | 1.6e-164 |
| AAK57187.1 | MxaC | BGC0001022 | Myxalamid\_biosynthetic\_gene\_cluster | 33 | 41.4953933381 | 578 | 4.8e-164 |
| BAG17643.1 | putative\_NRPS-type-I\_PKS\_fusion\_protein | BGC0001043 | SGR\_PTMs\_biosynthetic\_gene\_cluster | 34 | 41.1764705882 | 577 | 8.2e-164 |
| AAF19810.1 | MtaB | BGC0001024 | Myxothiazol\_biosynthetic\_gene\_cluster | 35 | 37.1367824238 | 576 | 2.4e-163 |
| CAQ18830.1 | polyketide\_synthase | BGC0000954 | Ajudazol\_biosynthetic\_gene\_cluster | 33 | 42.629340893 | 574 | 6.9e-163 |
| CCE88378.1 | polyketide\_synthase | BGC0001034 | Pellasoren\_biosynthetic\_gene\_cluster | 32 | 45.1807228916 | 574 | 6.9e-163 |
| AAS98783.1 | JamL | BGC0001001 | Jamaicamide\_biosynthetic\_gene\_cluster | 33 | 42.8419560595 | 572 | 2e-162 |
| BAN19720.1 | polyketide\_synthase | BGC0001252 | ACR-toxin\_biosynthetic\_gene\_cluster | 34 | 40.9284195606 | 572 | 3.4e-162 |
| CAG28678.1 | polyketide\_synthase | BGC0001023 | Myxochromide\_biosynthetic\_gene\_cluster | 32 | 42.9482636428 | 571 | 4.5e-162 |
| AAS98777.1 | JamE | BGC0001001 | Jamaicamide\_biosynthetic\_gene\_cluster | 37 | 33.0262225372 | 571 | 7.7e-162 |
| EFL02193.1 | amino\_acid\_adenylation\_domain-containing\_protein | BGC0000996 | Frontalamides\_biosynthetic\_gene\_cluster | 34 | 41.2473423104 | 571 | 7.7e-162 |
| CAQ43075.1 | polyketide\_synthase | BGC0000970 | Chondrochloren\_biosynthetic\_gene\_cluster | 32 | 45.7831325301 | 569 | 1.7e-161 |
| CCE88380.1 | polyketide\_synthase | BGC0001034 | Pellasoren\_biosynthetic\_gene\_cluster | 32 | 42.629340893 | 568 | 3.8e-161 |
| ABX60162.1 | polyketide\_synthase | BGC0000978 | Cylindrospermopsin\_biosynthetic\_gene\_cluster | 32 | 42.5230333097 | 566 | 2.5e-160 |
| ADF88280.1 | polyketide\_synthase | BGC0000981 | Cylindrospermopsin\_biosynthetic\_gene\_cluster | 32 | 42.5230333097 | 565 | 3.2e-160 |
| CBD77748.1 | polyketide\_synthase | BGC0000974 | Crocacin\_biosynthetic\_gene\_cluster | 34 | 41.6017009213 | 564 | 5.5e-160 |
| CCE88379.1 | polyketide\_synthase | BGC0001034 | Pellasoren\_biosynthetic\_gene\_cluster | 32 | 41.849751949 | 564 | 9.4e-160 |
| AMB48442.1 | polyketide\_synthase | BGC0001357 | Carbamidocyclophane\_biosynthetic\_gene\_cluster | 37 | 32.6718639263 | 559 | 3e-158 |
| AGC45620.1 | polyketide\_synthase | BGC0001394 | Phenalamide\_biosynthetic\_gene\_cluster | 31 | 45.2515946137 | 555 | 2.5e-157 |
| CAJ46690.1 | polyketide\_synthase | BGC0000969 | Chondramide\_biosynthetic\_gene\_cluster | 33 | 42.7710843373 | 553 | 1.3e-156 |
| CAJ46689.1 | polyketide\_synthase | BGC0000969 | Chondramide\_biosynthetic\_gene\_cluster | 32 | 43.5152374203 | 550 | 1.4e-155 |
| BAF02922.1 | type\_I\_polyketide\_synthase | BGC0000073 | Halstoctacosanolide\_biosynthetic\_gene\_cluster | 33 | 42.239546421 | 548 | 4.1e-155 |
| AIT55260.1 | GulC | BGC0000072 | Gulmirecin\_biosynthetic\_gene\_cluster | 32 | 44.968107725 | 548 | 5.3e-155 |
| AAK57189.1 | MxaE | BGC0001022 | Myxalamid\_biosynthetic\_gene\_cluster | 32 | 42.4167257264 | 547 | 9.1e-155 |
| AHA12078.1 | polyketide\_synthase\_type\_1 | BGC0001172 | Chlorizidine\_A\_biosynthetic\_gene\_cluster | 36 | 32.9907866761 | 545 | 3.4e-154 |
| ACY13414.1 | amino\_acid\_adenylation\_domain\_protein | BGC0001367 | Haliamide\_biosynthetic\_gene\_cluster | 31 | 45.1452870305 | 545 | 3.4e-154 |
| CAD19087.1 | StiC\_protein | BGC0000153 | Stigmatellin\_biosynthetic\_gene\_cluster | 33 | 43.5152374203 | 545 | 4.5e-154 |
| AEU11005.1 | NpnA | BGC0001029 | Nostophycin\_biosynthetic\_gene\_cluster | 36 | 32.6718639263 | 544 | 7.7e-154 |
| XP\_001220460.1 | hypothetical\_protein | BGC0001182 | Chaetoglobosins\_biosynthetic\_gene\_cluster | 37 | 31.9631467045 | 544 | 7.7e-154 |
| ANI24099.1 | polyketide\_synthase | BGC0001235 | Antalid\_biosynthetic\_gene\_cluster | 32 | 41.2119064493 | 542 | 2.9e-153 |
| AAO62584.1 | polyketide\_synthase\_type\_1 | BGC0001016 | Microcystin\_biosynthetic\_gene\_cluster | 33 | 42.8419560595 | 542 | 3.8e-153 |
| BAJ16467.1 | polyketide\_synthase | BGC0000058 | FD-891\_biosynthetic\_gene\_cluster | 33 | 35.8256555634 | 542 | 3.8e-153 |
| CAD19089.1 | StiE\_protein | BGC0000153 | Stigmatellin\_biosynthetic\_gene\_cluster | 36 | 32.7781715096 | 541 | 6.5e-153 |
| AFV96138.1 | polyketide\_synthase | BGC0001064 | Cylindrocyclophane\_biosynthetic\_gene\_cluster | 36 | 32.6718639263 | 541 | 6.5e-153 |
| ALD82522.1 | polyketide\_synthase | BGC0001212 | Nannocystin\_biosynthetic\_gene\_cluster | 32 | 42.0269312544 | 540 | 8.5e-153 |
| ADZ24998.1 | polyketide\_synthase | BGC0000380 | Leupyrrin\_biosynthetic\_gene\_cluster | 32 | 41.9560595322 | 540 | 1.4e-152 |
| ADF88275.1 | polyketide\_synthase | BGC0000981 | Cylindrospermopsin\_biosynthetic\_gene\_cluster | 36 | 32.4946846208 | 540 | 1.4e-152 |
| AAT70107.1 | CurL | BGC0001165 | Curacin\_biosynthetic\_gene\_cluster | 36 | 33.2034018427 | 539 | 1.9e-152 |
| AEE88278.1 | CurL | BGC0000976 | Curacin\_biosynthetic\_gene\_cluster | 36 | 33.2034018427 | 539 | 1.9e-152 |
| ABI94379.1 | tautomycetin\_biosynthetic\_PKS | BGC0000157 | Tautomycetin\_biosynthetic\_gene\_cluster | 32 | 42.629340893 | 539 | 2.5e-152 |
| AAW03328.1 | CtaE | BGC0000982 | Cystothiazole\_A\_biosynthetic\_gene\_cluster | 36 | 32.8844790928 | 539 | 2.5e-152 |
| ABV91286.1 | type\_I\_modular\_polyketide\_synthase | BGC0000158 | Tautomycetin\_biosynthetic\_gene\_cluster | 32 | 42.629340893 | 538 | 4.2e-152 |
| WP\_042799407.1 | polyketide\_synthase | BGC0001283 | Arsenopolyketides\_biosynthetic\_gene\_cluster | 35 | 38.2352941176 | 538 | 5.5e-152 |
| ACN69990.1 | polyketide\_synthase | BGC0000079 | Indanomycin\_biosynthetic\_gene\_cluster | 32 | 41.459957477 | 538 | 5.5e-152 |
| AEE88284.1 | CurF | BGC0000976 | Curacin\_biosynthetic\_gene\_cluster | 30 | 44.7200566974 | 537 | 9.4e-152 |
| AAY42396.1 | HctD | BGC0001000 | Hectochlorin\_biosynthetic\_gene\_cluster | 36 | 32.8844790928 | 537 | 1.2e-151 |
| CBD77732.1 | polyketide\_synthase | BGC0000974 | Crocacin\_biosynthetic\_gene\_cluster | 36 | 33.734939759 | 536 | 1.6e-151 |
| CAQ34928.1 | polyketide\_synthase | BGC0000986 | Dkxanthene\_biosynthetic\_gene\_cluster | 31 | 46.137491141 | 536 | 2.1e-151 |
| AKD43522.1 | Type\_I\_polyketide\_synthase | BGC0001409 | Dutomycin\_biosynthetic\_gene\_cluster | 35 | 35.6130403969 | 536 | 2.1e-151 |
| CAD19086.1 | StiB\_protein | BGC0000153 | Stigmatellin\_biosynthetic\_gene\_cluster | 35 | 32.565556343 | 536 | 2.1e-151 |
| ABX60153.1 | polyketide\_synthase | BGC0000978 | Cylindrospermopsin\_biosynthetic\_gene\_cluster | 36 | 32.4946846208 | 535 | 2.7e-151 |
| ADH04641.1 | TgaC | BGC0001051 | Thuggacin\_biosynthetic\_gene\_cluster | 32 | 40.6449326719 | 535 | 2.7e-151 |
| ADB12493.1 | EpoF | BGC0000990 | Epothilone\_biosynthetic\_gene\_cluster | 32 | 42.3104181432 | 535 | 2.7e-151 |
| CAQ34919.1 | polyketide\_synthase | BGC0000986 | Dkxanthene\_biosynthetic\_gene\_cluster | 33 | 42.4167257264 | 535 | 3.6e-151 |
| AAT70101.1 | CurF | BGC0001165 | Curacin\_biosynthetic\_gene\_cluster | 30 | 44.7200566974 | 535 | 3.6e-151 |
| AAF19813.1 | MtaE | BGC0001024 | Myxothiazol\_biosynthetic\_gene\_cluster | 35 | 33.7703756201 | 534 | 6.1e-151 |
| CAD89776.1 | MelE\_protein | BGC0001010 | Melithiazol\_biosynthetic\_gene\_cluster | 36 | 32.8136073707 | 534 | 6.1e-151 |
| AAF62885.1 | EpoF | BGC0000991 | Epothilone\_biosynthetic\_gene\_cluster | 32 | 42.3104181432 | 534 | 7.9e-151 |
| ADH04660.1 | TugD | BGC0001342 | Thuggacin\_biosynthetic\_gene\_cluster | 33 | 41.3182140326 | 534 | 7.9e-151 |
| AAF26923.1 | polyketide\_synthase | BGC0000988 | Epothilone\_biosynthetic\_gene\_cluster | 32 | 42.3458540043 | 534 | 1e-150 |
| ADB12490.1 | EpoC | BGC0000990 | Epothilone\_biosynthetic\_gene\_cluster | 32 | 41.6725726435 | 533 | 1.4e-150 |
| CAQ18828.1 | polyketide\_synthase | BGC0000954 | Ajudazol\_biosynthetic\_gene\_cluster | 33 | 42.7002126152 | 533 | 1.8e-150 |
| AAF26920.1 | polyketide\_synthase | BGC0000988 | Epothilone\_biosynthetic\_gene\_cluster | 32 | 41.6725726435 | 533 | 1.8e-150 |
| ACB46197.1 | polyketide\_synthase | BGC0000989 | Epothilone\_biosynthetic\_gene\_cluster | 32 | 42.3104181432 | 532 | 2.3e-150 |
| AAF62882.1 | EpoC | BGC0000991 | Epothilone\_biosynthetic\_gene\_cluster | 32 | 41.7080085046 | 532 | 3e-150 |
| CAQ18834.1 | polyketide\_synthase | BGC0000954 | Ajudazol\_biosynthetic\_gene\_cluster | 37 | 33.2388377038 | 531 | 5.1e-150 |
| ADZ24997.1 | polyketide\_synthase | BGC0000380 | Leupyrrin\_biosynthetic\_gene\_cluster | 30 | 45.3224663359 | 531 | 5.1e-150 |
| AAZ94388.1 | nodular\_polyketide\_synthase | BGC0000040 | Concanamycin\_A\_biosynthetic\_gene\_cluster | 32 | 43.1963146704 | 530 | 8.8e-150 |
| CAQ34920.1 | polyketide\_synthase | BGC0000986 | Dkxanthene\_biosynthetic\_gene\_cluster | 32 | 41.7434443657 | 530 | 8.8e-150 |
| AGC45623.1 | polyketide\_synthase | BGC0001394 | Phenalamide\_biosynthetic\_gene\_cluster | 31 | 42.7356484763 | 530 | 1.1e-149 |
| AAW03325.1 | CtaB | BGC0000982 | Cystothiazole\_A\_biosynthetic\_gene\_cluster | 36 | 33.2034018427 | 530 | 1.5e-149 |
| CAL58684.1 | polyketide\_synthase | BGC0000149 | Spirangien\_biosynthetic\_gene\_cluster | 33 | 37.3139617293 | 529 | 2e-149 |
| CUW01171.1 | Type\_I\_polyketide\_synthase | BGC0001359 | PM100117\_/\_PM100118\_biosynthetic\_gene\_cluster | 37 | 33.0616583983 | 529 | 2.6e-149 |
| CAO98850.1 | polyketide\_synthase\_AufG | BGC0000023 | Aurafuron\_biosynthetic\_gene\_cluster | 32 | 40.9992912828 | 529 | 2.6e-149 |
| AAF00959.1 | McyD | BGC0001017 | Microcystin\_biosynthetic\_gene\_cluster | 31 | 41.6017009213 | 529 | 3.3e-149 |
| CBD77736.1 | polyketide\_synthase | BGC0000974 | Crocacin\_biosynthetic\_gene\_cluster | 31 | 45.8540042523 | 528 | 4.4e-149 |
| BAF02927.1 | type\_I\_polyketide\_synthase | BGC0000073 | Halstoctacosanolide\_biosynthetic\_gene\_cluster | 34 | 36.4635010631 | 527 | 7.4e-149 |
| AAS98781.1 | JamJ | BGC0001001 | Jamaicamide\_biosynthetic\_gene\_cluster | 30 | 42.629340893 | 527 | 9.7e-149 |
| AHA12079.1 | polyketide\_synthase\_type\_1 | BGC0001172 | Chlorizidine\_A\_biosynthetic\_gene\_cluster | 36 | 32.9199149539 | 527 | 9.7e-149 |
| AGC45622.1 | polyketide\_synthase | BGC0001394 | Phenalamide\_biosynthetic\_gene\_cluster | 32 | 41.1410347271 | 527 | 9.7e-149 |
| CAQ18833.1 | polyketide\_synthase | BGC0000954 | Ajudazol\_biosynthetic\_gene\_cluster | 35 | 33.2742735648 | 526 | 1.7e-148 |
| AAO62582.1 | polyketide\_synthase\_peptide\_sythetase\_fusion\_protein | BGC0001016 | Microcystin\_biosynthetic\_gene\_cluster | 36 | 32.9907866761 | 526 | 1.7e-148 |
| AIT55262.1 | GulE | BGC0000072 | Gulmirecin\_biosynthetic\_gene\_cluster | 36 | 32.565556343 | 526 | 2.2e-148 |
| CAO98849.1 | polyketide\_synthase\_AufF | BGC0000023 | Aurafuron\_biosynthetic\_gene\_cluster | 35 | 33.6640680369 | 525 | 3.7e-148 |
| AEZ64503.1 | Herd | BGC0001065 | Herboxidiene\_biosynthetic\_gene\_cluster | 35 | 36.4635010631 | 525 | 3.7e-148 |
| ACR33079.1 | polyketide\_synthase | BGC0000017 | Anatoxin\_biosynthetic\_gene\_cluster | 34 | 32.8490432318 | 525 | 4.8e-148 |
| CCE88381.1 | polyketide\_synthase | BGC0001034 | Pellasoren\_biosynthetic\_gene\_cluster | 32 | 41.849751949 | 524 | 6.3e-148 |
| ABK32255.1 | AmbA | BGC0000014 | Ambruticin\_biosynthetic\_gene\_cluster | 36 | 33.5577604536 | 524 | 8.2e-148 |
| AIW82282.1 | PuwE | BGC0001125 | Puwainaphycins\_biosynthetic\_gene\_cluster | 35 | 33.2034018427 | 524 | 8.2e-148 |
| CAD29793.1 | polyketide\_synthase\_type\_I | BGC0001015 | Microcystin\_biosynthetic\_gene\_cluster | 31 | 41.849751949 | 524 | 1.1e-147 |
| ADM46358.1 | polyketide\_synthase | BGC0000106 | Naphthomycin\_biosynthetic\_gene\_cluster | 33 | 41.6371367824 | 524 | 1.1e-147 |
| ACB46194.1 | polyketide\_synthase | BGC0000989 | Epothilone\_biosynthetic\_gene\_cluster | 32 | 41.6725726435 | 523 | 1.4e-147 |
| CAQ18835.1 | polyketide\_synthase | BGC0000954 | Ajudazol\_biosynthetic\_gene\_cluster | 33 | 42.0978029766 | 523 | 1.4e-147 |
| CAQ43078.1 | polyketide\_synthase | BGC0000970 | Chondrochloren\_biosynthetic\_gene\_cluster | 32 | 43.408929837 | 522 | 3.1e-147 |
| AEE88281.1 | CurI | BGC0000976 | Curacin\_biosynthetic\_gene\_cluster | 35 | 33.1325301205 | 522 | 3.1e-147 |
| ABW96541.1 | type\_I\_modular\_polyketide\_synthase | BGC0000159 | Tautomycin\_biosynthetic\_gene\_cluster | 32 | 39.8299078668 | 522 | 3.1e-147 |
| AAT70104.1 | CurI | BGC0001165 | Curacin\_biosynthetic\_gene\_cluster | 35 | 33.1325301205 | 522 | 3.1e-147 |
| AAQ82564.1 | FscC | BGC0000034 | Candicidin\_biosynthetic\_gene\_cluster | 33 | 40.6094968108 | 522 | 4.1e-147 |
| ABW96540.1 | type\_I\_modular\_polyketide\_synthase | BGC0000159 | Tautomycin\_biosynthetic\_gene\_cluster | 31 | 42.239546421 | 522 | 4.1e-147 |
| ABW96542.1 | type\_I\_modular\_polyketide\_synthase | BGC0000159 | Tautomycin\_biosynthetic\_gene\_cluster | 31 | 42.4167257264 | 521 | 5.3e-147 |
| AAK19883.1 | soraphen\_polyketide\_synthase\_A | BGC0000147 | Soraphen\_biosynthetic\_gene\_cluster | 30 | 45.7831325301 | 521 | 5.3e-147 |
| ABV97152.1 | Beta-ketoacyl\_synthase | BGC0000137 | Rifamycin\_biosynthetic\_gene\_cluster | 32 | 41.3536498937 | 521 | 5.3e-147 |
| AAF00958.1 | McyE | BGC0001017 | Microcystin\_biosynthetic\_gene\_cluster | 35 | 32.7427356485 | 521 | 7e-147 |
| ACN69988.1 | polyketide\_synthase | BGC0000079 | Indanomycin\_biosynthetic\_gene\_cluster | 33 | 36.2508858965 | 521 | 7e-147 |
| CUW01175.1 | Type\_I\_polyketide\_synthase | BGC0001359 | PM100117\_/\_PM100118\_biosynthetic\_gene\_cluster | 32 | 43.0900070872 | 520 | 1.2e-146 |
| ABB76806.1 | polyketide\_synthase\_2 | BGC0000155\_c2 | T-toxin\_biosynthetic\_gene\_cluster | 32 | 38.1644223955 | 520 | 1.5e-146 |
| AAF19814.1 | MtaF | BGC0001024 | Myxothiazol\_biosynthetic\_gene\_cluster | 35 | 32.8136073707 | 519 | 2e-146 |
| CAD19092.1 | StiH\_protein | BGC0000153 | Stigmatellin\_biosynthetic\_gene\_cluster | 36 | 32.6718639263 | 519 | 2e-146 |
| AEU11006.1 | NpnB | BGC0001029 | Nostophycin\_biosynthetic\_gene\_cluster | 35 | 35.0106307583 | 519 | 2e-146 |
| AHH34186.1 | polyketide\_synthase | BGC0001161 | 1-heptadecene\_biosynthetic\_gene\_cluster | 34 | 33.6995038979 | 519 | 2.6e-146 |
| BAJ16468.1 | polyketide\_synthase | BGC0000058 | FD-891\_biosynthetic\_gene\_cluster | 33 | 36.7824238129 | 518 | 4.5e-146 |
| CAD19085.1 | StiA\_protein | BGC0000153 | Stigmatellin\_biosynthetic\_gene\_cluster | 36 | 31.4316087881 | 518 | 4.5e-146 |
| AHB82064.1 | polyketide\_synthase | BGC0001231 | Microsclerodermins\_biosynthetic\_gene\_cluster | 36 | 32.5301204819 | 518 | 4.5e-146 |
| CAC20931.1 | PimS1\_protein | BGC0000125 | Pimaricin\_biosynthetic\_gene\_cluster | 34 | 37.4911410347 | 518 | 5.9e-146 |
| AAC01711.1 | RifB | BGC0000136 | Rifamycin\_biosynthetic\_gene\_cluster | 31 | 40.2905740609 | 517 | 7.7e-146 |
| CAQ43077.1 | polyketide\_synthase | BGC0000970 | Chondrochloren\_biosynthetic\_gene\_cluster | 32 | 42.1332388377 | 517 | 7.7e-146 |
| ABK32287.1 | JerA | BGC0000080 | Jerangolid\_biosynthetic\_gene\_cluster | 35 | 32.7781715096 | 517 | 1e-145 |
| CAD29794.1 | peptide\_synthetase | BGC0001015 | Microcystin\_biosynthetic\_gene\_cluster | 35 | 32.8844790928 | 517 | 1e-145 |
| BAK64638.1 | polyketide\_synthase | BGC0000135 | Reveromycin\_biosynthetic\_gene\_cluster | 33 | 37.207654146 | 516 | 1.7e-145 |
| AID65222.1 | putative\_aspartate\_racemase | BGC0000335 | Cystomanamides\_biosynthetic\_gene\_cluster | 36 | 32.7427356485 | 516 | 1.7e-145 |
| BAF02923.1 | type\_I\_polyketide\_synthase | BGC0000073 | Halstoctacosanolide\_biosynthetic\_gene\_cluster | 32 | 41.7788802268 | 516 | 2.2e-145 |
| CAQ18829.1 | polyketide\_synthase | BGC0000954 | Ajudazol\_biosynthetic\_gene\_cluster | 31 | 42.5584691708 | 515 | 2.9e-145 |
| CQR60493.1 | Polyketide\_synthase,\_type\_I,\_modules:\_9\_and\_10 | BGC0001287 | Chaxamycin\_A\_/\_chaxamycin\_B\_/\_chaxamycin\_C\_/\_chaxamycin\_D | 33 | 41.3536498937 | 515 | 3.8e-145 |
| CAD19088.1 | StiD\_protein | BGC0000153 | Stigmatellin\_biosynthetic\_gene\_cluster | 35 | 33.2034018427 | 514 | 6.5e-145 |
| ACN69989.1 | polyketide\_synthase | BGC0000079 | Indanomycin\_biosynthetic\_gene\_cluster | 32 | 41.6371367824 | 514 | 6.5e-145 |
| CBD77734.1 | polyketide\_synthase | BGC0000974 | Crocacin\_biosynthetic\_gene\_cluster | 35 | 32.8844790928 | 514 | 6.5e-145 |
| ADM46357.1 | polyketide\_synthase | BGC0000106 | Naphthomycin\_biosynthetic\_gene\_cluster | 32 | 41.6017009213 | 514 | 8.5e-145 |
| ABB05104.1 | LipPks3 | BGC0001003 | Lipomycin\_biosynthetic\_gene\_cluster | 34 | 37.4911410347 | 514 | 1.1e-144 |
| BAJ16470.1 | polyketide\_synthase | BGC0000058 | FD-891\_biosynthetic\_gene\_cluster | 31 | 45.6413890858 | 513 | 1.9e-144 |
| AAK73514.1 | AmphC | BGC0000015 | Amphotericin\_biosynthetic\_gene\_cluster | 35 | 35.9319631467 | 512 | 2.5e-144 |
| BAJ16471.1 | polyketide\_synthase | BGC0000058 | FD-891\_biosynthetic\_gene\_cluster | 32 | 41.9206236712 | 512 | 4.2e-144 |
| CAD89773.1 | MelB\_protein | BGC0001009 | Melithiazol\_biosynthetic\_gene\_cluster | 35 | 32.9199149539 | 512 | 4.2e-144 |
| BAF85844.1 | modular\_polyketide\_synthase | BGC0000109 | Nemadectin\_biosynthetic\_gene\_cluster | 33 | 42.5939050319 | 512 | 4.2e-144 |
| AKD43753.1 | HerB | BGC0001349 | Heronamide\_biosynthetic\_gene\_cluster | 32 | 37.7037562013 | 511 | 5.5e-144 |
| ABO15860.1 | polyketide\_synthase | BGC0000130 | Pyrrolomycin\_biosynthetic\_gene\_cluster | 35 | 34.7271438696 | 511 | 5.5e-144 |
| ADZ24996.1 | polyketide\_synthase | BGC0000380 | Leupyrrin\_biosynthetic\_gene\_cluster | 31 | 42.8773919206 | 511 | 5.5e-144 |
| AEZ53949.1 | polyketide\_synthase | BGC0000144 | Salinomycin\_biosynthetic\_gene\_cluster | 32 | 41.7788802268 | 511 | 5.5e-144 |
| ADC79637.1 | TamAI | BGC0001052 | Tirandamycin\_biosynthetic\_gene\_cluster | 35 | 32.7427356485 | 511 | 7.2e-144 |
| AIT55259.1 | GulB | BGC0000072 | Gulmirecin\_biosynthetic\_gene\_cluster | 36 | 32.9907866761 | 511 | 7.2e-144 |
| AHH34189.1 | polyketide\_synthase | BGC0001162 | 1-heptadecene\_biosynthetic\_gene\_cluster | 34 | 34.1601700921 | 509 | 2.1e-143 |
| ADF88277.1 | polyketide\_synthase | BGC0000981 | Cylindrospermopsin\_biosynthetic\_gene\_cluster | 35 | 33.1325301205 | 509 | 2.1e-143 |
| BAF02926.1 | type\_I\_polyketide\_synthase | BGC0000073 | Halstoctacosanolide\_biosynthetic\_gene\_cluster | 35 | 33.5931963147 | 509 | 2.1e-143 |
| ADC45538.1 | modular\_polyketide\_synthase | BGC0000093 | Meilingmycin\_biosynthetic\_gene\_cluster | 32 | 35.648476258 | 509 | 2.7e-143 |
| BAQ21939.1 | putative\_type\_I\_polyketide\_synthase | BGC0001204 | Versipelostatin\_biosynthetic\_gene\_cluster | 32 | 42.5939050319 | 508 | 3.6e-143 |
| CAM00064.1 | EryAII\_Erythromycin\_polyketide\_synthase\_modules\_3\_and\_4 | BGC0000055 | Erythromycin\_biosynthetic\_gene\_cluster | 31 | 39.6527285613 | 508 | 3.6e-143 |
| AAK57186.1 | MxaB2 | BGC0001022 | Myxalamid\_biosynthetic\_gene\_cluster | 36 | 31.0772501772 | 508 | 4.7e-143 |
| EAU29808.1 | hypothetical\_protein | BGC0001400 | Citreoviridin\_biosynthetic\_gene\_cluster | 33 | 41.1410347271 | 508 | 4.7e-143 |
| BAQ21947.1 | putative\_type\_I\_polyketide\_synthase | BGC0001204 | Versipelostatin\_biosynthetic\_gene\_cluster | 32 | 40.5740609497 | 508 | 4.7e-143 |
| CAO98879.1 | polyketide\_synthase\_AufD | BGC0000023 | Aurafuron\_biosynthetic\_gene\_cluster | 33 | 35.0815024805 | 508 | 6.1e-143 |
| AEP40934.1 | polyketide\_synthase\_type\_I | BGC0000021 | Apoptolidin\_biosynthetic\_gene\_cluster | 31 | 41.6725726435 | 507 | 8e-143 |
| AAC01714.1 | RifE | BGC0000136 | Rifamycin\_biosynthetic\_gene\_cluster | 33 | 39.7944720057 | 507 | 1e-142 |
| BAC68129.1 | modular\_polyketide\_synthase | BGC0000059 | Filipin\_biosynthetic\_gene\_cluster | 35 | 33.2388377038 | 507 | 1e-142 |
| ABX60163.1 | polyketide\_synthase | BGC0000978 | Cylindrospermopsin\_biosynthetic\_gene\_cluster | 35 | 33.1325301205 | 507 | 1e-142 |
| AHH99926.1 | PKS\_I | BGC0000002 | Aculeximycin\_biosynthetic\_gene\_cluster | 30 | 45.4287739192 | 507 | 1.4e-142 |
| CAL58685.1 | polyketide\_synthase | BGC0000149 | Spirangien\_biosynthetic\_gene\_cluster | 34 | 32.565556343 | 506 | 1.8e-142 |
| AIT55261.1 | GulD | BGC0000072 | Gulmirecin\_biosynthetic\_gene\_cluster | 35 | 33.2742735648 | 506 | 1.8e-142 |
| BAJ16469.1 | polyketide\_synthase | BGC0000058 | FD-891\_biosynthetic\_gene\_cluster | 31 | 40.0425230333 | 506 | 1.8e-142 |
| CAD19093.1 | StiJ\_protein | BGC0000153 | Stigmatellin\_biosynthetic\_gene\_cluster | 35 | 33.734939759 | 506 | 1.8e-142 |
| ABJ97439.1 | MerC | BGC0001012 | Meridamycin\_biosynthetic\_gene\_cluster | 33 | 35.4004252303 | 506 | 1.8e-142 |
| CAL58681.1 | polyketide\_synthase | BGC0000149 | Spirangien\_biosynthetic\_gene\_cluster | 33 | 36.4989369242 | 506 | 2.3e-142 |
| ALA09371.1 | type\_I\_modular\_PKS | BGC0001303 | Lobosamide\_biosynthetic\_gene\_cluster | 32 | 41.2473423104 | 506 | 2.3e-142 |
| AEZ54379.1 | PieA6 | BGC0000124 | Piericidin\_A1\_biosynthetic\_gene\_cluster | 35 | 33.1325301205 | 505 | 3e-142 |
| AFU82616.1 | polyketide\_synthase | BGC0000998 | Guadinomine\_biosynthetic\_gene\_cluster | 32 | 39.4755492558 | 505 | 5.2e-142 |
| ABP55210.1 | beta-ketoacyl\_synthase | BGC0000142 | Salinilactam\_biosynthetic\_gene\_cluster | 33 | 36.2508858965 | 505 | 5.2e-142 |
| CAL58687.1 | polyketide\_synthase | BGC0000149 | Spirangien\_biosynthetic\_gene\_cluster | 32 | 41.9560595322 | 505 | 5.2e-142 |
| BAB69196.1 | modular\_polyketide\_synthase | BGC0000117 | Oligomycin\_biosynthetic\_gene\_cluster | 32 | 40.5386250886 | 505 | 5.2e-142 |
| AEZ54378.1 | PieA5 | BGC0000124 | Piericidin\_A1\_biosynthetic\_gene\_cluster | 34 | 36.3926293409 | 505 | 5.2e-142 |
| BAQ21948.1 | putative\_type\_I\_polyketide\_synthase | BGC0001204 | Versipelostatin\_biosynthetic\_gene\_cluster | 34 | 32.8844790928 | 504 | 6.7e-142 |
| ABB05105.1 | LipPks4 | BGC0001003 | Lipomycin\_biosynthetic\_gene\_cluster | 31 | 42.3812898653 | 503 | 1.1e-141 |
| CQR60496.1 | Polyketide\_synthase,\_type\_I,\_modules:\_4,\_5\_and\_6 | BGC0001287 | Chaxamycin\_A\_/\_chaxamycin\_B\_/\_chaxamycin\_C\_/\_chaxamycin\_D | 32 | 41.5308291991 | 503 | 1.1e-141 |
| AEU17899.1 | putative\_type\_I\_PKS | BGC0001072 | Simocyclinone\_biosynthetic\_gene\_cluster | 34 | 32.9199149539 | 503 | 2e-141 |
| ctg1\_orf23 |  | BGC0001013 | Meridamycin\_biosynthetic\_gene\_cluster | 33 | 35.5067328136 | 503 | 2e-141 |
| ABC87512.1 | polyketide\_synthase | BGC0001011 | Meridamycin\_biosynthetic\_gene\_cluster | 33 | 35.5067328136 | 503 | 2e-141 |
| AAG23262.1 | polyketide\_synthase\_extender\_modules\_8-10 | BGC0000148 | Spinosad\_biosynthetic\_gene\_cluster | 33 | 35.4004252303 | 502 | 4.4e-141 |
| BAQ21946.1 | putative\_type\_I\_polyketide\_synthase | BGC0001204 | Versipelostatin\_biosynthetic\_gene\_cluster | 30 | 46.2083628632 | 502 | 4.4e-141 |
| ABV91287.1 | type\_I\_modular\_polyketide\_synthase | BGC0000158 | Tautomycetin\_biosynthetic\_gene\_cluster | 33 | 37.0659107016 | 501 | 7.4e-141 |
| ACO94456.1 | polyketide\_synthase\_type\_I | BGC0000029 | BE-14106\_biosynthetic\_gene\_cluster | 33 | 36.1091424522 | 501 | 7.4e-141 |
| CAD89777.1 | MelF\_protein | BGC0001009 | Melithiazol\_biosynthetic\_gene\_cluster | 34 | 32.8844790928 | 500 | 9.7e-141 |
| BAQ21940.1 | putative\_Type\_I\_polyketide\_synthase | BGC0001204 | Versipelostatin\_biosynthetic\_gene\_cluster | 32 | 40.2197023388 | 500 | 9.7e-141 |
| CUW01177.1 | Type\_I\_polyketide\_synthase | BGC0001359\_c2 | PM100117\_/\_PM100118\_biosynthetic\_gene\_cluster | 32 | 41.3890857548 | 500 | 9.7e-141 |
| ABI94380.1 | tautomycetin\_biosynthetic\_PKS | BGC0000157 | Tautomycetin\_biosynthetic\_gene\_cluster | 33 | 37.1367824238 | 500 | 1.3e-140 |
| BAC57028.1 | protomycinolide\_IV\_synthase\_1 | BGC0000102 | Mycinamicin\_biosynthetic\_gene\_cluster | 34 | 32.4946846208 | 500 | 1.3e-140 |
| ABV97155.1 | Acyl\_transferase | BGC0000137 | Rifamycin\_biosynthetic\_gene\_cluster | 33 | 40.0070871722 | 500 | 1.7e-140 |
| AFV30247.1 | polyketide\_synthase | BGC0000075 | Hygrocin\_biosynthetic\_gene\_cluster | 33 | 36.428065202 | 499 | 2.2e-140 |
| AFV96142.1 | polyketide\_synthase | BGC0001064 | Cylindrocyclophane\_biosynthetic\_gene\_cluster | 33 | 33.3097094259 | 499 | 2.8e-140 |
| CAQ64691.1 | lasalocid\_modular\_polyketide\_synthase | BGC0000087 | Lasalocid\_biosynthetic\_gene\_cluster | 32 | 41.3890857548 | 499 | 2.8e-140 |
| CAQ18832.1 | polyketide\_synthase | BGC0000954 | Ajudazol\_biosynthetic\_gene\_cluster | 32 | 42.8065201984 | 499 | 2.8e-140 |
| AAZ77693.1 | ChlA1 | BGC0000036 | Chlorothricin\_biosynthetic\_gene\_cluster | 36 | 32.8490432318 | 499 | 2.8e-140 |
| AFV30250.1 | polyketide\_synthase | BGC0000075 | Hygrocin\_biosynthetic\_gene\_cluster | 31 | 41.4953933381 | 498 | 3.7e-140 |
| AEP40940.1 | polyketide\_synthase\_type\_I | BGC0000021 | Apoptolidin\_biosynthetic\_gene\_cluster | 35 | 32.3883770376 | 498 | 4.8e-140 |
| BAK64650.1 | polyketide\_synthase | BGC0000135 | Reveromycin\_biosynthetic\_gene\_cluster | 34 | 36.3217576187 | 498 | 4.8e-140 |
| BAT51066.1 | type\_I\_polyketide\_synthase | BGC0001296 | Streptazone\_E\_biosynthetic\_gene\_cluster | 31 | 42.8065201984 | 498 | 6.3e-140 |
| ABB05103.1 | LipPks2 | BGC0001003 | Lipomycin\_biosynthetic\_gene\_cluster | 32 | 42.0978029766 | 498 | 6.3e-140 |
| AHN85651.1 | Phn2 | BGC0000122 | Phenylnannolone\_A\_biosynthetic\_gene\_cluster | 30 | 42.3812898653 | 498 | 6.3e-140 |
| AHB82053.1 | polyketide\_synthase | BGC0001019 | Microsclerodermins\_biosynthetic\_gene\_cluster | 35 | 32.6364280652 | 498 | 6.3e-140 |
| AAZ94386.1 | modular\_polyketide\_synthase | BGC0000040 | Concanamycin\_A\_biosynthetic\_gene\_cluster | 32 | 43.0545712261 | 497 | 8.2e-140 |
| ADN13832.1 | Beta-ketoacyl\_synthase | BGC0001164 | 1-heptadecene\_biosynthetic\_gene\_cluster | 34 | 34.0538625089 | 497 | 1.1e-139 |
| BAO98805.1 | putative\_polyketide\_synthase | BGC0001002 | Jawsamycin\_biosynthetic\_gene\_cluster | 34 | 32.9907866761 | 497 | 1.1e-139 |
| CAD55506.1 | putative\_type\_I\_polyketide\_synthase | BGC0000038 | Coelimycin\_biosynthetic\_gene\_cluster | 30 | 46.137491141 | 497 | 1.1e-139 |
| ACF35445.1 | MbcAI | BGC0000090 | Macbecin\_biosynthetic\_gene\_cluster | 33 | 36.428065202 | 497 | 1.1e-139 |
| BAG85031.1 | putative\_polyketide\_synthase | BGC0000086 | Lasalocid\_biosynthetic\_gene\_cluster | 32 | 41.6371367824 | 497 | 1.4e-139 |
| AFU82617.1 | polyketide\_synthase | BGC0000998 | Guadinomine\_biosynthetic\_gene\_cluster | 36 | 32.4592487597 | 497 | 1.4e-139 |
| ADX66472.1 | ScnS1 | BGC0000108 | Natamycin\_biosynthetic\_gene\_cluster | 33 | 38.2707299787 | 497 | 1.4e-139 |
| AEH42491.1 | polyketide\_synthase | BGC0000032 | Calcimycin\_biosynthetic\_gene\_cluster | 32 | 36.4989369242 | 496 | 1.8e-139 |
| ADM46359.1 | polyketide\_synthase | BGC0000106 | Naphthomycin\_biosynthetic\_gene\_cluster | 33 | 36.1091424522 | 496 | 2.4e-139 |
| CAE46843.1 | Type\_I\_modular\_polyketide\_synthase | BGC0000103 | Mycolactone\_biosynthetic\_gene\_cluster | 35 | 33.345145287 | 496 | 2.4e-139 |
| CAQ52626.1 | type\_I\_polyketide\_synthase,\_loading\_module\_and\_modules\_1-3 | BGC0001066 | Kendomycin\_biosynthetic\_gene\_cluster | 35 | 34.1601700921 | 495 | 3.1e-139 |
| AEZ54377.1 | PieA4 | BGC0000124 | Piericidin\_A1\_biosynthetic\_gene\_cluster | 34 | 35.5067328136 | 495 | 3.1e-139 |
| AAS98782.1 | JamK | BGC0001001 | Jamaicamide\_biosynthetic\_gene\_cluster | 35 | 30.5811481219 | 495 | 4.1e-139 |
| BAQ25513.1 | type\_I\_polyketide\_synthase | BGC0001288 | Maklamicin\_biosynthetic\_gene\_cluster | 31 | 45.1807228916 | 495 | 4.1e-139 |
| ACB37755.1 | putative\_type\_I\_polyketide\_synthase | BGC0000162 | Tetrocarcin\_A\_biosynthetic\_gene\_cluster | 32 | 41.6017009213 | 495 | 4.1e-139 |
| CAE46851.1 | Type\_I\_modular\_polyketide\_synthase | BGC0000103 | Mycolactone\_biosynthetic\_gene\_cluster | 31 | 42.2041105599 | 495 | 4.1e-139 |
| AEZ64505.1 | Herb | BGC0001065 | Herboxidiene\_biosynthetic\_gene\_cluster | 32 | 42.1332388377 | 495 | 4.1e-139 |
| CAQ64686.1 | lasalocid\_modular\_polyketide\_synthase | BGC0000087 | Lasalocid\_biosynthetic\_gene\_cluster | 31 | 42.2041105599 | 495 | 5.3e-139 |
| ctg1\_orf11 |  | BGC0000053 | Elaiophylin\_biosynthetic\_gene\_cluster | 33 | 36.1445783133 | 494 | 7e-139 |
| BAG85026.1 | putative\_polyketide\_synthase | BGC0000086 | Lasalocid\_biosynthetic\_gene\_cluster | 31 | 42.2041105599 | 494 | 7e-139 |
| CAL58682.1 | polyketide\_synthase | BGC0000149 | Spirangien\_biosynthetic\_gene\_cluster | 34 | 35.6130403969 | 494 | 7e-139 |
| CBD77738.1 | polyketide\_synthase | BGC0000974 | Crocacin\_biosynthetic\_gene\_cluster | 35 | 33.1679659816 | 494 | 7e-139 |
| ADM46356.1 | polyketide\_synthase | BGC0000106 | Naphthomycin\_biosynthetic\_gene\_cluster | 33 | 35.1523742027 | 493 | 1.2e-138 |
| AEP40939.1 | polyketide\_synthase\_type\_I | BGC0000021 | Apoptolidin\_biosynthetic\_gene\_cluster | 31 | 41.3536498937 | 493 | 1.2e-138 |
| AEC13069.1 | fosC | BGC0000060 | Fostriecin\_biosynthetic\_gene\_cluster | 34 | 37.6328844791 | 493 | 1.2e-138 |
| AAO65798.1 | monensin\_polyketide\_synthase\_modules\_3\_and\_4 | BGC0000100 | Monensin\_biosynthetic\_gene\_cluster | 31 | 42.8773919206 | 493 | 1.6e-138 |
| CAQ64687.1 | lasalocid\_modular\_polyketide\_synthase | BGC0000087 | Lasalocid\_biosynthetic\_gene\_cluster | 32 | 37.0659107016 | 493 | 1.6e-138 |
| BAG85027.1 | putative\_polyketide\_synthase | BGC0000086 | Lasalocid\_biosynthetic\_gene\_cluster | 32 | 37.0659107016 | 493 | 1.6e-138 |
| AAW03329.1 | CtaF | BGC0000982 | Cystothiazole\_A\_biosynthetic\_gene\_cluster | 34 | 32.8136073707 | 493 | 1.6e-138 |
| BAT51065.1 | type\_I\_polyketide\_synthase | BGC0001296 | Streptazone\_E\_biosynthetic\_gene\_cluster | 32 | 36.3571934798 | 493 | 1.6e-138 |
| AGI99496.1 | Type\_I\_polyketide\_synthase | BGC0001004 | Lobophorin\_biosynthetic\_gene\_cluster | 32 | 41.6017009213 | 493 | 1.6e-138 |
| AAO65806.1 | monensin\_polyketide\_synthase\_modules\_11\_and\_12 | BGC0000100 | Monensin\_biosynthetic\_gene\_cluster | 33 | 36.3571934798 | 493 | 2e-138 |
| ctg1\_orf10 |  | BGC0000053 | Elaiophylin\_biosynthetic\_gene\_cluster | 31 | 41.8851878101 | 493 | 2e-138 |
| ABC84456.1 | NigAI | BGC0000114 | Nigericin\_biosynthetic\_gene\_cluster | 35 | 33.0616583983 | 492 | 2.6e-138 |
| AAK57190.1 | MxaF | BGC0001022 | Myxalamid\_biosynthetic\_gene\_cluster | 35 | 31.5024805103 | 492 | 2.6e-138 |
| ABV83230.1 | CppC | BGC0000116 | Nystatin-like\_Pseudonocardia\_polyene\_biosynthetic\_gene\_cluster | 34 | 36.1445783133 | 492 | 2.6e-138 |
| CUW01172.1 | Type\_I\_polyketide\_synthase | BGC0001359 | PM100117\_/\_PM100118\_biosynthetic\_gene\_cluster | 34 | 32.8844790928 | 492 | 2.6e-138 |
| AAQ82567.1 | FscE | BGC0000034 | Candicidin\_biosynthetic\_gene\_cluster | 32 | 37.9518072289 | 492 | 3.5e-138 |
| BAB69192.1 | modular\_polyketide\_synthase | BGC0000117 | Oligomycin\_biosynthetic\_gene\_cluster | 32 | 42.7002126152 | 492 | 4.5e-138 |
| CAQ18838.1 | polyketide\_synthase | BGC0000954 | Ajudazol\_biosynthetic\_gene\_cluster | 37 | 31.0772501772 | 492 | 4.5e-138 |
| AAZ77698.1 | ChlA5 | BGC0000036 | Chlorothricin\_biosynthetic\_gene\_cluster | 32 | 41.4245216159 | 491 | 5.9e-138 |
| ADH04639.1 | TgaA | BGC0001051 | Thuggacin\_biosynthetic\_gene\_cluster | 36 | 31.2898653437 | 491 | 5.9e-138 |
| CQR60494.1 | Polyketide\_synthase,\_type\_I,\_module\_8 | BGC0001287 | Chaxamycin\_A\_/\_chaxamycin\_B\_/\_chaxamycin\_C\_/\_chaxamycin\_D | 32 | 38.0581148122 | 491 | 5.9e-138 |
| AAF71775.1 | NysB | BGC0000115 | Nystatin\_biosynthetic\_gene\_cluster | 34 | 33.9475549256 | 491 | 7.7e-138 |
| CAN89636.1 | putative\_polyketide\_synthase | BGC0001070 | Kirromycin\_biosynthetic\_gene\_cluster | 33 | 35.0106307583 | 491 | 7.7e-138 |
| ACO94468.1 | polyketide\_synthase\_type\_I | BGC0000029 | BE-14106\_biosynthetic\_gene\_cluster | 33 | 36.3217576187 | 490 | 1e-137 |
| AAQ82568.1 | FscD | BGC0000034 | Candicidin\_biosynthetic\_gene\_cluster | 34 | 33.4160170092 | 490 | 1e-137 |
| AEC13070.1 | fosD | BGC0000060 | Fostriecin\_biosynthetic\_gene\_cluster | 34 | 33.1679659816 | 490 | 1.3e-137 |
| AAC46025.1 | polyketide\_synthase\_module\_3 | BGC0000113 | Niddamycin\_biosynthetic\_gene\_cluster | 32 | 41.3890857548 | 490 | 1.7e-137 |
| ACB46487.1 | polyketide\_synthase | BGC0000082 | Kijanimicin\_biosynthetic\_gene\_cluster | 32 | 41.8143160879 | 490 | 1.7e-137 |
| ABK32289.1 | JerC | BGC0000080 | Jerangolid\_biosynthetic\_gene\_cluster | 32 | 38.4479092842 | 489 | 2.2e-137 |
| WP\_043476516.1 | beta-ketoacyl\_synthase | BGC0001232 | Leucanicidin\_biosynthetic\_gene\_cluster | 32 | 36.3926293409 | 489 | 2.2e-137 |
| AEZ53946.1 | polyketide\_synthase | BGC0000144 | Salinomycin\_biosynthetic\_gene\_cluster | 32 | 36.03827073 | 489 | 2.9e-137 |
| BAD08359.1 | polyketide\_synthase\_modules\_5-6 | BGC0000167 | Vicenistatin\_biosynthetic\_gene\_cluster | 31 | 41.5662650602 | 488 | 3.8e-137 |
| ACO94470.1 | polyketide\_synthase\_type\_I | BGC0000029 | BE-14106\_biosynthetic\_gene\_cluster | 31 | 41.4953933381 | 488 | 3.8e-137 |
| AAC01712.2 | RifC | BGC0000136 | Rifamycin\_biosynthetic\_gene\_cluster | 31 | 39.9007795889 | 488 | 3.8e-137 |
| ACB46488.1 | polyketide\_synthase | BGC0000082 | Kijanimicin\_biosynthetic\_gene\_cluster | 34 | 32.8136073707 | 488 | 5e-137 |
| AIT55264.1 | GulA | BGC0000072 | Gulmirecin\_biosynthetic\_gene\_cluster | 34 | 31.6796598157 | 488 | 5e-137 |
| AAX98191.1 | polyketide\_synthase\_type\_I | BGC0000052 | ECO-02301\_biosynthetic\_gene\_cluster | 31 | 42.4167257264 | 488 | 5e-137 |
| AAG23266.1 | polyketide\_synthase\_extender\_modules\_3-4 | BGC0000148 | Spinosad\_biosynthetic\_gene\_cluster | 34 | 32.8490432318 | 488 | 5e-137 |
| AAG23265.1 | polyketide\_synthase\_extender\_module\_2 | BGC0000148 | Spinosad\_biosynthetic\_gene\_cluster | 31 | 39.93621545 | 488 | 6.5e-137 |
| AGI99497.1 | type\_I\_polyketide\_synthase | BGC0001004 | Lobophorin\_biosynthetic\_gene\_cluster | 35 | 33.1679659816 | 488 | 6.5e-137 |
| ACN64831.1 | PokM1 | BGC0001061 | Polyketomycin\_biosynthetic\_gene\_cluster | 33 | 36.1091424522 | 488 | 6.5e-137 |
| ABK32257.1 | AmbC | BGC0000014 | Ambruticin\_biosynthetic\_gene\_cluster | 32 | 36.1800141743 | 487 | 8.5e-137 |
| CAO98852.1 | polyketide\_synthase\_AufI | BGC0000023 | Aurafuron\_biosynthetic\_gene\_cluster | 35 | 31.2189936215 | 487 | 8.5e-137 |
| AGY30677.1 | Ann5 | BGC0001298 | 4-Z-annimycin\_biosynthetic\_gene\_cluster | 31 | 43.3026222537 | 487 | 8.5e-137 |
| AAQ84156.1 | Plm1 | BGC0000123 | Phoslactomycin\_B\_biosynthetic\_gene\_cluster | 31 | 45.357902197 | 487 | 1.1e-136 |
| CQR60495.1 | Polyketide\_synthase,\_type\_I,\_module\_7 | BGC0001287 | Chaxamycin\_A\_/\_chaxamycin\_B\_/\_chaxamycin\_C\_/\_chaxamycin\_D | 33 | 35.1523742027 | 487 | 1.1e-136 |
| AAF71776.1 | NysC | BGC0000115 | Nystatin\_biosynthetic\_gene\_cluster | 33 | 36.817859674 | 487 | 1.1e-136 |
| ABC84469.1 | NigAIX | BGC0000114 | Nigericin\_biosynthetic\_gene\_cluster | 32 | 36.817859674 | 487 | 1.5e-136 |
| AGI61658.1 | modular\_PKS | BGC0000095\_c3 | Micromonolactam\_biosynthetic\_gene\_cluster | 31 | 44.3302622254 | 487 | 1.5e-136 |
| AAZ77696.1 | ChlA3 | BGC0000036 | Chlorothricin\_biosynthetic\_gene\_cluster | 31 | 39.8299078668 | 486 | 2.5e-136 |
| BAE93730.1 | type\_I\_polyketide\_synthase | BGC0000164 | Tetronomycin\_biosynthetic\_gene\_cluster | 30 | 46.7399007796 | 486 | 2.5e-136 |
| AGY30676.1 | Ann4 | BGC0001298 | 4-Z-annimycin\_biosynthetic\_gene\_cluster | 32 | 41.1055988661 | 485 | 3.2e-136 |
| AEH42490.1 | polyketide\_synthase | BGC0000032 | Calcimycin\_biosynthetic\_gene\_cluster | 34 | 32.5301204819 | 485 | 3.2e-136 |
| ctg1\_orf7 |  | BGC0000053 | Elaiophylin\_biosynthetic\_gene\_cluster | 35 | 32.3175053154 | 485 | 4.2e-136 |
| AAQ82566.1 | FscF | BGC0000034 | Candicidin\_biosynthetic\_gene\_cluster | 34 | 35.0815024805 | 485 | 4.2e-136 |
| AGC09484.1 | LobS1 | BGC0001183 | Lobophorin\_biosynthetic\_gene\_cluster | 34 | 33.4868887314 | 485 | 4.2e-136 |
| ctg1\_orf254 |  | BGC0001200 | Lorneic\_acid\_A\_biosynthetic\_gene\_cluster | 31 | 41.5308291991 | 485 | 4.2e-136 |
| CAE45669.1 | borrelidin\_polyketide\_synthase,\_type\_I | BGC0000031 | Borrelidin\_biosynthetic\_gene\_cluster | 33 | 35.1523742027 | 485 | 4.2e-136 |
| CAE46850.1 | Type\_I\_modular\_polyketide\_synthase | BGC0000103 | Mycolactone\_biosynthetic\_gene\_cluster | 31 | 42.1332388377 | 485 | 5.5e-136 |
| ACO94483.1 | polyketide\_synthase\_type\_I | BGC0000097 | ML-449\_biosynthetic\_gene\_cluster | 35 | 32.7781715096 | 485 | 5.5e-136 |
| ACB46471.1 | polyketide\_synthase | BGC0000082 | Kijanimicin\_biosynthetic\_gene\_cluster | 33 | 36.4635010631 | 485 | 5.5e-136 |
| CAE02606.1 | polyketide\_synthase\_type\_I | BGC0000024 | Aureothin\_biosynthetic\_gene\_cluster | 33 | 33.0616583983 | 485 | 5.5e-136 |
| AMK48247.1 | 6-methylsalicylic\_acid\_synthase | BGC0001351\_c2 | Cahuitamycins\_biosynthetic\_gene\_cluster | 33 | 36.3217576187 | 485 | 5.5e-136 |
| ALP32043.1 | CycC | BGC0001293 | Cyclizidine\_biosynthetic\_gene\_cluster | 32 | 38.6250885897 | 484 | 7.2e-136 |
| AEZ64504.1 | Herc | BGC0001065 | Herboxidiene\_biosynthetic\_gene\_cluster | 31 | 39.8653437279 | 484 | 7.2e-136 |
| CUW01174.1 | Type\_I\_polyketide\_synthase | BGC0001359 | PM100117\_/\_PM100118\_biosynthetic\_gene\_cluster | 32 | 36.6406803685 | 484 | 7.2e-136 |
| BAF02921.1 | type\_I\_polyketide\_synthase | BGC0000073 | Halstoctacosanolide\_biosynthetic\_gene\_cluster | 34 | 32.9199149539 | 484 | 9.4e-136 |
| ctg1\_orf521 |  | BGC0001199 | Akaeolide\_biosynthetic\_gene\_cluster | 31 | 42.5939050319 | 484 | 9.4e-136 |
| AAZ94390.1 | modular\_polyketide\_synthase | BGC0000040 | Concanamycin\_A\_biosynthetic\_gene\_cluster | 30 | 41.2119064493 | 484 | 9.4e-136 |
| ALA09357.1 | type\_I\_modular\_PKS | BGC0001303 | Lobosamide\_biosynthetic\_gene\_cluster | 31 | 44.3302622254 | 484 | 9.4e-136 |
| ADX66461.1 | ScnS2 | BGC0000108 | Natamycin\_biosynthetic\_gene\_cluster | 31 | 41.9560595322 | 483 | 1.2e-135 |
| ABB88534.1 | polyketide\_synthase\_type\_I | BGC0000049 | E-492\_/\_E-975\_biosynthetic\_gene\_cluster | 32 | 39.93621545 | 483 | 1.2e-135 |
| ADH04640.1 | TgaB | BGC0001051 | Thuggacin\_biosynthetic\_gene\_cluster | 34 | 32.6718639263 | 483 | 1.2e-135 |
| AAK73502.1 | AmphJ | BGC0000015 | Amphotericin\_biosynthetic\_gene\_cluster | 32 | 36.5343727853 | 483 | 1.2e-135 |
| AAD03047.1 | type\_I\_polyketide\_synthase | BGC0000041 | Coronafacic\_acid\_biosynthetic\_gene\_cluster | 33 | 36.8887313962 | 483 | 1.2e-135 |
| CUW01173.1 | Type\_I\_polyketide\_synthase | BGC0001359 | PM100117\_/\_PM100118\_biosynthetic\_gene\_cluster | 31 | 41.6371367824 | 483 | 1.6e-135 |
| AAQ84157.1 | Plm2-3 | BGC0000123 | Phoslactomycin\_B\_biosynthetic\_gene\_cluster | 33 | 36.2508858965 | 483 | 1.6e-135 |
| ctg1\_orf29 |  | BGC0000096 | Midecamycin\_biosynthetic\_gene\_cluster | 34 | 32.5301204819 | 483 | 1.6e-135 |
| BAD08360.1 | polyketide\_synthase\_modules\_7-8 | BGC0000167 | Vicenistatin\_biosynthetic\_gene\_cluster | 31 | 41.7788802268 | 483 | 1.6e-135 |
| ADC79618.1 | BafAIII | BGC0000028 | Bafilomycin\_biosynthetic\_gene\_cluster | 33 | 35.4004252303 | 483 | 1.6e-135 |
| AAC38075.1 | polyketide\_synthase\_type\_I | BGC0000127 | Pyoluteorin\_biosynthetic\_gene\_cluster | 34 | 32.6718639263 | 483 | 2.1e-135 |
| AGY62755.1 | EbeC | BGC0000051 | Ebelactone\_biosynthetic\_gene\_cluster | 32 | 33.1679659816 | 483 | 2.1e-135 |
| ABC84471.1 | NigAVII | BGC0000114 | Nigericin\_biosynthetic\_gene\_cluster | 33 | 34.2310418143 | 483 | 2.1e-135 |
| BAB69194.1 | modular\_polyketide\_synthase | BGC0000117 | Oligomycin\_biosynthetic\_gene\_cluster | 30 | 41.9560595322 | 482 | 2.7e-135 |
| ALJ49921.1 | TtmH | BGC0001236 | Thiotetroamide\_biosynthetic\_gene\_cluster | 30 | 41.7434443657 | 482 | 3.6e-135 |
| ABC84457.1 | NigAII | BGC0000114 | Nigericin\_biosynthetic\_gene\_cluster | 32 | 36.3571934798 | 482 | 3.6e-135 |
| AFL48526.1 | laidlomycin\_polyketide\_synthase\_(module\_2) | BGC0000084 | Laidlomycin\_biosynthetic\_gene\_cluster | 32 | 36.3217576187 | 482 | 4.7e-135 |
| ACB37740.1 | putative\_type\_I\_polyketide\_synthase | BGC0000162 | Tetrocarcin\_A\_biosynthetic\_gene\_cluster | 30 | 41.459957477 | 482 | 4.7e-135 |
| ctg1\_orf28 |  | BGC0000096 | Midecamycin\_biosynthetic\_gene\_cluster | 32 | 38.0935506733 | 482 | 4.7e-135 |
| AAG13918.1 | megalomicin\_6-deoxyerythronolide\_B\_synthase\_2 | BGC0000092 | Megalomicin\_biosynthetic\_gene\_cluster | 31 | 35.9319631467 | 482 | 4.7e-135 |
| ABJ97438.1 | MerB | BGC0001012 | Meridamycin\_biosynthetic\_gene\_cluster | 32 | 36.817859674 | 482 | 4.7e-135 |
| ACO94499.1 | polyketide\_synthase\_type\_I | BGC0000097 | ML-449\_biosynthetic\_gene\_cluster | 31 | 41.3890857548 | 482 | 4.7e-135 |
| CCP20050.1 | divL3\_protein | BGC0001119 | Divergolide\_biosynthetic\_gene\_cluster | 32 | 37.3493975904 | 481 | 6.1e-135 |
| ABV97151.1 | AMP-dependent\_synthetase\_and\_ligase | BGC0000137 | Rifamycin\_biosynthetic\_gene\_cluster | 33 | 36.7115520907 | 481 | 6.1e-135 |
| ABC87510.1 | polyketide\_synthase | BGC0001011 | Meridamycin\_biosynthetic\_gene\_cluster | 32 | 36.6761162296 | 481 | 6.1e-135 |
| ctg1\_orf21 |  | BGC0001013 | Meridamycin\_biosynthetic\_gene\_cluster | 32 | 36.6761162296 | 481 | 6.1e-135 |
| AAK73513.1 | AmphB | BGC0000015 | Amphotericin\_biosynthetic\_gene\_cluster | 33 | 33.6286321758 | 481 | 6.1e-135 |
| AEZ54375.1 | PieA2 | BGC0000124 | Piericidin\_A1\_biosynthetic\_gene\_cluster | 32 | 36.3571934798 | 481 | 8e-135 |
| AAK73501.1 | AmphI | BGC0000015 | Amphotericin\_biosynthetic\_gene\_cluster | 34 | 32.7427356485 | 481 | 8e-135 |
| AAP85335.1 | type\_I\_PKS | BGC0000233 | Hedamycin\_biosynthetic\_gene\_cluster | 33 | 37.0304748405 | 480 | 1e-134 |
| AAC46028.1 | polyketide\_synthase\_module\_7 | BGC0000113 | Niddamycin\_biosynthetic\_gene\_cluster | 33 | 33.345145287 | 480 | 1.4e-134 |
| ACY06286.1 | polyketide\_synthase | BGC0001042 | Sanglifehrin\_A\_biosynthetic\_gene\_cluster | 32 | 35.2941176471 | 480 | 1.8e-134 |
| ACB37741.1 | putative\_type\_I\_polyketide\_synthase | BGC0000162 | Tetrocarcin\_A\_biosynthetic\_gene\_cluster | 30 | 41.8851878101 | 480 | 1.8e-134 |
| AKD43761.1 | HerD | BGC0001349 | Heronamide\_biosynthetic\_gene\_cluster | 32 | 35.9673990078 | 480 | 1.8e-134 |
| AFL48525.1 | laidlomycin\_polyketide\_synthase\_(loading\_module\_and\_module\_1) | BGC0000084 | Laidlomycin\_biosynthetic\_gene\_cluster | 35 | 33.3097094259 | 480 | 1.8e-134 |
| AEZ53945.1 | polyketide\_synthase | BGC0000144 | Salinomycin\_biosynthetic\_gene\_cluster | 35 | 33.1679659816 | 479 | 2.3e-134 |
| AAC69329.1 | type\_I\_polyketide\_synthase\_PikAI | BGC0000094 | Methymycin\_/\_pikromycin\_biosynthetic\_gene\_cluster | 33 | 33.8058114812 | 479 | 2.3e-134 |
| ABK32263.1 | AmbH | BGC0000014 | Ambruticin\_biosynthetic\_gene\_cluster | 35 | 33.4514528703 | 479 | 2.3e-134 |
| AFL48532.1 | laidlomycin\_polyketide\_synthase\_(module\_11\_and\_module\_12) | BGC0000084 | Laidlomycin\_biosynthetic\_gene\_cluster | 32 | 35.4358610914 | 479 | 2.3e-134 |
| ADX66470.1 | ScnS0 | BGC0000108 | Natamycin\_biosynthetic\_gene\_cluster | 34 | 33.0262225372 | 479 | 3e-134 |
| AMB48441.1 | polyketide\_synthase | BGC0001357 | Carbamidocyclophane\_biosynthetic\_gene\_cluster | 33 | 32.5301204819 | 479 | 3e-134 |
| AHH99921.1 | PKS\_I | BGC0000002 | Aculeximycin\_biosynthetic\_gene\_cluster | 33 | 37.4911410347 | 479 | 3e-134 |
| CUI25744.1 | Polyketide\_synthase/Nonribosomal\_peptide\_synthase | BGC0001354 | U-68204\_biosynthetic\_gene\_cluster | 29 | 45.747696669 | 479 | 3e-134 |
| CAL58686.1 | polyketide\_synthase | BGC0000149 | Spirangien\_biosynthetic\_gene\_cluster | 32 | 37.8809355067 | 479 | 3e-134 |
| ABK32258.1 | AmbD | BGC0000014 | Ambruticin\_biosynthetic\_gene\_cluster | 32 | 36.7115520907 | 478 | 4e-134 |
| BAB69195.1 | modular\_polyketide\_synthase | BGC0000117 | Oligomycin\_biosynthetic\_gene\_cluster | 34 | 33.2388377038 | 478 | 4e-134 |
| AAC46026.1 | polyketide\_synthase\_modules\_4\_and\_5 | BGC0000113 | Niddamycin\_biosynthetic\_gene\_cluster | 34 | 32.9199149539 | 478 | 4e-134 |
| AAY89049.1 | polyketide\_synthase | BGC0001069 | Chivosazole\_biosynthetic\_gene\_cluster | 36 | 31.6796598157 | 478 | 4e-134 |
| AAU04878.1 | polyketide\_synthase | BGC0000365 | Glycopeptidolipid\_biosynthetic\_gene\_cluster | 33 | 33.8412473423 | 478 | 5.2e-134 |
| BAD38874.1 | polyketide\_synthase | BGC0000111 | Neocarzilin\_biosynthetic\_gene\_cluster | 32 | 42.4167257264 | 478 | 6.7e-134 |
| CAI94713.1 | putative\_polyketide\_synthase | BGC0000141 | Rubradirin\_biosynthetic\_gene\_cluster | 30 | 41.4245216159 | 478 | 6.7e-134 |
| CAC20921.1 | PimS2\_protein | BGC0000125 | Pimaricin\_biosynthetic\_gene\_cluster | 34 | 33.5577604536 | 477 | 8.8e-134 |
| WP\_048832936.1 | polyketide\_synthase | BGC0001348 | JBIR-100\_biosynthetic\_gene\_cluster | 31 | 42.9128277817 | 477 | 8.8e-134 |
| CAC20930.1 | PimS0\_protein | BGC0000125 | Pimaricin\_biosynthetic\_gene\_cluster | 35 | 32.175761871 | 477 | 8.8e-134 |
| AAF19812.1 | MtaD | BGC0001024 | Myxothiazol\_biosynthetic\_gene\_cluster | 30 | 43.6215450035 | 477 | 8.8e-134 |
| BAD08373.1 | polyketide\_synthase\_modules\_1-3 | BGC0000167 | Vicenistatin\_biosynthetic\_gene\_cluster | 30 | 41.6017009213 | 477 | 8.8e-134 |
| BAF02924.1 | type\_I\_polyketide\_synthase | BGC0000073 | Halstoctacosanolide\_biosynthetic\_gene\_cluster | 33 | 33.3097094259 | 477 | 1.2e-133 |
| CQR60497.1 | Polyketide\_synthase,\_type\_I,\_modules:\_loading,\_1,\_2\_and\_3 | BGC0001287 | Chaxamycin\_A\_/\_chaxamycin\_B\_/\_chaxamycin\_C\_/\_chaxamycin\_D | 33 | 36.8532955351 | 477 | 1.2e-133 |
| ADC79639.1 | TamAIII | BGC0001052 | Tirandamycin\_biosynthetic\_gene\_cluster | 30 | 45.357902197 | 477 | 1.5e-133 |
| ABK32290.1 | JerD | BGC0000080 | Jerangolid\_biosynthetic\_gene\_cluster | 31 | 40.1842664777 | 476 | 2e-133 |
| ACY06289.1 | type\_I\_polyketide\_synthase | BGC0001042 | Sanglifehrin\_A\_biosynthetic\_gene\_cluster | 31 | 41.3182140326 | 476 | 2.6e-133 |
| ABV97153.1 | Beta-ketoacyl\_synthase | BGC0000137 | Rifamycin\_biosynthetic\_gene\_cluster | 31 | 41.3890857548 | 476 | 2.6e-133 |
| ctg1\_orf256 |  | BGC0001200 | Lorneic\_acid\_A\_biosynthetic\_gene\_cluster | 30 | 46.137491141 | 476 | 2.6e-133 |
| AAM54075.1 | polyketide\_synthase | BGC0000020 | Ansamitocin\_biosynthetic\_gene\_cluster | 34 | 35.9673990078 | 475 | 3.3e-133 |
| BAK64649.1 | polyketide\_synthase | BGC0000135 | Reveromycin\_biosynthetic\_gene\_cluster | 33 | 36.1800141743 | 475 | 3.3e-133 |
| CAC22144.1 | putative\_type\_I\_polyketide\_synthase | BGC0000038 | Coelimycin\_biosynthetic\_gene\_cluster | 32 | 36.3217576187 | 475 | 3.3e-133 |
| BAC57032.1 | protomycinolide\_IV\_synthase\_5 | BGC0000102 | Mycinamicin\_biosynthetic\_gene\_cluster | 30 | 42.0623671155 | 475 | 5.7e-133 |
| CCE88377.1 | non-ribosomal\_peptide\_synthetase/polyketide\_synthase | BGC0001034 | Pellasoren\_biosynthetic\_gene\_cluster | 35 | 31.5379163714 | 475 | 5.7e-133 |
| AFV30248.1 | polyketide\_synthase | BGC0000075 | Hygrocin\_biosynthetic\_gene\_cluster | 32 | 37.7037562013 | 474 | 7.5e-133 |
| AAG23264.1 | polyketide\_synthase\_loading\_and\_extender\_module\_1 | BGC0000148 | Spinosad\_biosynthetic\_gene\_cluster | 34 | 32.7427356485 | 474 | 9.7e-133 |
| CBA11584.1 | polyketide\_synthase\_type\_I | BGC0001046 | Streptolydigin\_biosynthetic\_gene\_cluster | 34 | 33.4514528703 | 474 | 9.7e-133 |
| CCP20047.1 | divK\_protein | BGC0001119 | Divergolide\_biosynthetic\_gene\_cluster | 34 | 32.7781715096 | 474 | 9.7e-133 |
| AAF26919.1 | polyketide\_synthase | BGC0000988 | Epothilone\_biosynthetic\_gene\_cluster | 34 | 32.955350815 | 474 | 9.7e-133 |
| CAQ52624.1 | type\_I\_polyketide\_synthase,\_modules\_7-8 | BGC0001066 | Kendomycin\_biosynthetic\_gene\_cluster | 32 | 36.3571934798 | 474 | 9.7e-133 |
| BAO66529.1 | type\_I\_polyketide\_synthase | BGC0000042 | Cremimycin\_biosynthetic\_gene\_cluster | 33 | 36.6406803685 | 474 | 9.7e-133 |
| ABV97154.1 | Beta-ketoacyl\_synthase | BGC0000137 | Rifamycin\_biosynthetic\_gene\_cluster | 30 | 40.0779588944 | 474 | 9.7e-133 |
| CAC20920.1 | PimS3\_protein | BGC0000125 | Pimaricin\_biosynthetic\_gene\_cluster | 33 | 36.7469879518 | 473 | 1.3e-132 |
| AAX98186.1 | polyketide\_synthase\_type\_I | BGC0000052 | ECO-02301\_biosynthetic\_gene\_cluster | 32 | 36.3926293409 | 473 | 1.3e-132 |
| ADH04657.1 | TugA | BGC0001342 | Thuggacin\_biosynthetic\_gene\_cluster | 36 | 31.6087880936 | 473 | 1.3e-132 |
| ABP55223.1 | beta-ketoacyl\_synthase | BGC0000142 | Salinilactam\_biosynthetic\_gene\_cluster | 33 | 36.8532955351 | 473 | 1.3e-132 |
| AAC69330.1 | type\_I\_polyketide\_synthase\_PikAII | BGC0000094 | Methymycin\_/\_pikromycin\_biosynthetic\_gene\_cluster | 32 | 36.3571934798 | 473 | 1.7e-132 |
| CAJ88176.1 | putative\_polyketide\_synthase\_B | BGC0000151 | Stambomycin\_biosynthetic\_gene\_cluster | 33 | 36.2863217576 | 473 | 1.7e-132 |
| AHH99919.1 | PKS\_I | BGC0000002 | Aculeximycin\_biosynthetic\_gene\_cluster | 32 | 36.9950389794 | 473 | 1.7e-132 |
| ACB46192.1 | polyketide\_synthase | BGC0000989 | Epothilone\_biosynthetic\_gene\_cluster | 34 | 32.955350815 | 473 | 2.2e-132 |
| AAF62880.1 | EpoA | BGC0000991 | Epothilone\_biosynthetic\_gene\_cluster | 34 | 32.955350815 | 473 | 2.2e-132 |
| BAD38873.1 | polyketide\_synthase | BGC0000111 | Neocarzilin\_biosynthetic\_gene\_cluster | 34 | 35.1878100638 | 473 | 2.2e-132 |
| AAP42859.1 | NanA5 | BGC0000105 | Nanchangmycin\_biosynthetic\_gene\_cluster | 29 | 45.8540042523 | 473 | 2.2e-132 |
| AAF71766.1 | NysI | BGC0000115 | Nystatin\_biosynthetic\_gene\_cluster | 34 | 32.7427356485 | 472 | 2.8e-132 |
| ANC94964.1 | AlmHIII | BGC0001396 | Aldgamycin\_biosynthetic\_gene\_cluster | 31 | 36.3571934798 | 472 | 2.8e-132 |
| AFV30251.1 | polyketide\_synthase | BGC0000075 | Hygrocin\_biosynthetic\_gene\_cluster | 30 | 42.3458540043 | 472 | 3.7e-132 |
| AAM70355.1 | CalO5 | BGC0000033 | Calicheamicin\_biosynthetic\_gene\_cluster | 33 | 35.3295535082 | 472 | 3.7e-132 |
| ACY06290.1 | type\_I\_polyketide\_synthase | BGC0001042 | Sanglifehrin\_A\_biosynthetic\_gene\_cluster | 34 | 33.8412473423 | 472 | 4.8e-132 |
| ADB12488.1 | EpoA | BGC0000990 | Epothilone\_biosynthetic\_gene\_cluster | 34 | 32.955350815 | 472 | 4.8e-132 |
| AAM81586.2 | putative\_type\_I\_polyketide\_synthase | BGC0000047 | Dihydrochalcomycin\_biosynthetic\_gene\_cluster | 31 | 36.3217576187 | 472 | 4.8e-132 |
| ABC84470.1 | NIGAVIII | BGC0000114 | Nigericin\_biosynthetic\_gene\_cluster | 30 | 40.1133947555 | 472 | 4.8e-132 |
| AAS79461.1 | polyketide\_synthase\_subunit | BGC0000035 | Chalcomycin\_biosynthetic\_gene\_cluster | 33 | 32.9907866761 | 472 | 4.8e-132 |
| ACO94496.1 | polyketide\_synthase\_type\_I | BGC0000097 | ML-449\_biosynthetic\_gene\_cluster | 32 | 36.3217576187 | 471 | 6.3e-132 |
| AGI99482.1 | Type\_I\_polyketide\_synthase | BGC0001004 | Lobophorin\_biosynthetic\_gene\_cluster | 32 | 38.0581148122 | 471 | 6.3e-132 |
| AKD43765.1 | HerE | BGC0001349 | Heronamide\_biosynthetic\_gene\_cluster | 33 | 35.6839121191 | 471 | 6.3e-132 |
| ANC94966.1 | AlmHI | BGC0001396 | Aldgamycin\_biosynthetic\_gene\_cluster | 34 | 32.6718639263 | 471 | 8.2e-132 |
| ACY06288.1 | type\_I\_polyketide\_synthase | BGC0001042 | Sanglifehrin\_A\_biosynthetic\_gene\_cluster | 31 | 41.4245216159 | 471 | 8.2e-132 |
| CAJ88184.1 | putative\_modular\_polyketide\_synthase | BGC0000151 | Stambomycin\_biosynthetic\_gene\_cluster | 35 | 33.6640680369 | 471 | 8.2e-132 |
| AGC09499.1 | LobS4 | BGC0001183 | Lobophorin\_biosynthetic\_gene\_cluster | 32 | 38.0581148122 | 471 | 8.2e-132 |
| CAC22145.1 | putative\_type\_I\_polyketide\_synthase | BGC0000038 | Coelimycin\_biosynthetic\_gene\_cluster | 30 | 41.8143160879 | 471 | 8.2e-132 |
| CUW01176.1 | Type\_I\_polyketide\_synthase | BGC0001359\_c2 | PM100117\_/\_PM100118\_biosynthetic\_gene\_cluster | 34 | 32.9199149539 | 470 | 1.1e-131 |
| AEU17897.1 | putative\_type\_I\_PKS | BGC0001072 | Simocyclinone\_biosynthetic\_gene\_cluster | 32 | 35.8256555634 | 470 | 1.1e-131 |
| ctg1\_orf522 |  | BGC0001199 | Akaeolide\_biosynthetic\_gene\_cluster | 32 | 37.5265768958 | 470 | 1.1e-131 |
| CAJ88186.1 | putative\_modular\_polyketide\_synthase | BGC0000151 | Stambomycin\_biosynthetic\_gene\_cluster | 30 | 45.1452870305 | 470 | 1.1e-131 |
| AAO06918.1 | GdmAIII | BGC0000066 | Geldanamycin\_biosynthetic\_gene\_cluster | 30 | 41.7434443657 | 470 | 1.4e-131 |
| ABB86410.1 | GelC | BGC0000067 | Geldanamycin\_biosynthetic\_gene\_cluster | 30 | 41.7434443657 | 470 | 1.4e-131 |
| CAE45668.1 | borrelidin\_polyketide\_synthase,\_type\_I | BGC0000031 | Borrelidin\_biosynthetic\_gene\_cluster | 34 | 32.6718639263 | 470 | 1.4e-131 |
| BAG85028.1 | putative\_polyketide\_synthase | BGC0000086 | Lasalocid\_biosynthetic\_gene\_cluster | 33 | 33.1679659816 | 470 | 1.4e-131 |
| ACY06287.1 | type\_I\_polyketide\_synthase | BGC0001042 | Sanglifehrin\_A\_biosynthetic\_gene\_cluster | 33 | 34.5854004252 | 470 | 1.4e-131 |
| AEU17898.1 | putative\_type\_I\_PKS | BGC0001072 | Simocyclinone\_biosynthetic\_gene\_cluster | 33 | 32.955350815 | 470 | 1.4e-131 |
| CAQ64688.1 | lasalocid\_modular\_polyketide\_synthase | BGC0000087 | Lasalocid\_biosynthetic\_gene\_cluster | 33 | 33.1679659816 | 470 | 1.4e-131 |
| ADX66459.1 | ScnS4 | BGC0000108 | Natamycin\_biosynthetic\_gene\_cluster | 32 | 36.5698086464 | 470 | 1.4e-131 |
| BAG85029.1 | putative\_polyketide\_synthase | BGC0000086 | Lasalocid\_biosynthetic\_gene\_cluster | 34 | 32.955350815 | 470 | 1.4e-131 |
| AFU82615.1 | polyketide\_synthase | BGC0000998 | Guadinomine\_biosynthetic\_gene\_cluster | 35 | 32.4946846208 | 470 | 1.4e-131 |
| ABB88523.1 | polyketide\_synthase\_type\_I | BGC0000050 | E-837\_biosynthetic\_gene\_cluster | 34 | 33.0262225372 | 470 | 1.8e-131 |
| AMY15057.1 | tetraketide\_synthase\_MF-SQTKS | BGC0001339 | Squalestatin\_S1\_biosynthetic\_gene\_cluster | 36 | 30.7583274274 | 470 | 1.8e-131 |
| ACR50774.1 | polyketide\_synthase | BGC0000163 | Tetronasin\_biosynthetic\_gene\_cluster | 30 | 41.6017009213 | 469 | 2.4e-131 |
| AAC01710.1 | RifA | BGC0000136 | Rifamycin\_biosynthetic\_gene\_cluster | 33 | 32.9907866761 | 469 | 2.4e-131 |
| ABJ97437.1 | MerA | BGC0001012 | Meridamycin\_biosynthetic\_gene\_cluster | 33 | 33.5223245925 | 469 | 2.4e-131 |
| AGY62758.1 | EbeF | BGC0000051 | Ebelactone\_biosynthetic\_gene\_cluster | 30 | 41.4953933381 | 469 | 2.4e-131 |
| AAQ84147.1 | Plm7 | BGC0000123 | Phoslactomycin\_B\_biosynthetic\_gene\_cluster | 35 | 32.6364280652 | 469 | 3.1e-131 |
| ABK32288.1 | JerB | BGC0000080 | Jerangolid\_biosynthetic\_gene\_cluster | 32 | 36.428065202 | 469 | 3.1e-131 |
| BAE93731.1 | type\_I\_polyketide\_synthase | BGC0000164 | Tetronomycin\_biosynthetic\_gene\_cluster | 30 | 41.6371367824 | 469 | 3.1e-131 |
| AAY28227.1 | HbmAIII | BGC0000074 | Herbimycin\_biosynthetic\_gene\_cluster | 30 | 41.8851878101 | 469 | 3.1e-131 |
| CAQ64689.1 | lasalocid\_modular\_polyketide\_synthase | BGC0000087 | Lasalocid\_biosynthetic\_gene\_cluster | 34 | 32.955350815 | 469 | 3.1e-131 |
| CAO85897.1 | modular\_polyketide\_synthase\_NorB | BGC0000110 | Neoaureothin\_biosynthetic\_gene\_cluster | 32 | 35.6839121191 | 468 | 4.1e-131 |
| AAS79462.1 | polyketide\_synthase\_subunit | BGC0000035 | Chalcomycin\_biosynthetic\_gene\_cluster | 33 | 32.9199149539 | 468 | 5.3e-131 |
| AFP87523.1 | type\_I\_polyketide\_synthase | BGC0001159 | Marinopyrrole\_biosynthetic\_gene\_cluster | 33 | 32.6364280652 | 468 | 5.3e-131 |
| CAO85896.1 | protein\_modular\_polyketide\_synthase\_NorA' | BGC0000110 | Neoaureothin\_biosynthetic\_gene\_cluster | 31 | 41.7788802268 | 468 | 5.3e-131 |
| AHA38203.1 | GphJ | BGC0000069 | Gephyronic\_acid\_biosynthetic\_gene\_cluster | 35 | 32.7781715096 | 468 | 5.3e-131 |
| AKD43769.1 | HerA2 | BGC0001349 | Heronamide\_biosynthetic\_gene\_cluster | 32 | 35.5067328136 | 468 | 5.3e-131 |
| BAE93729.1 | type\_I\_polyketide\_synthase | BGC0000164 | Tetronomycin\_biosynthetic\_gene\_cluster | 31 | 36.2508858965 | 468 | 5.3e-131 |
| AAU93805.2 | polyketide\_synthase\_modules\_5\_and\_6 | BGC0000054 | Erythromycin\_biosynthetic\_gene\_cluster | 32 | 35.1523742027 | 468 | 5.3e-131 |
| BAF85843.1 | modular\_polyketide\_synthase | BGC0000109 | Nemadectin\_biosynthetic\_gene\_cluster | 31 | 42.4875974486 | 468 | 7e-131 |
| ALP32042.1 | CycB | BGC0001293 | Cyclizidine\_biosynthetic\_gene\_cluster | 32 | 36.3571934798 | 468 | 7e-131 |
| AAZ77697.1 | ChlA4 | BGC0000036 | Chlorothricin\_biosynthetic\_gene\_cluster | 32 | 40.0070871722 | 468 | 7e-131 |
| ctg1\_orf27 |  | BGC0000096 | Midecamycin\_biosynthetic\_gene\_cluster | 33 | 34.3019135365 | 468 | 7e-131 |
| CUI25702.1 | Tu3010\_Hybrid\_PKS-NRPS\_system | BGC0001352 | Thiotetronate\_Tu\_3010\_biosynthetic\_gene\_cluster | 28 | 45.2515946137 | 468 | 7e-131 |
| ADC79620.1 | BafAV | BGC0000028 | Bafilomycin\_biosynthetic\_gene\_cluster | 32 | 35.1878100638 | 468 | 7e-131 |
| AAG23263.1 | polyketide\_synthase\_extender\_modules\_5-7 | BGC0000148 | Spinosad\_biosynthetic\_gene\_cluster | 34 | 32.8844790928 | 467 | 9.1e-131 |
| AKD43763.1 | HerG | BGC0001349 | Heronamide\_biosynthetic\_gene\_cluster | 33 | 35.5067328136 | 467 | 9.1e-131 |
| CAO85893.1 | modular\_polyketide\_synthase\_NorA | BGC0000110 | Neoaureothin\_biosynthetic\_gene\_cluster | 32 | 36.3571934798 | 467 | 9.1e-131 |
| ABV83229.1 | CppB | BGC0000116 | Nystatin-like\_Pseudonocardia\_polyene\_biosynthetic\_gene\_cluster | 34 | 32.7781715096 | 467 | 9.1e-131 |
| AAB66506.1 | tylactone\_synthase\_modules\_4\_&\_5 | BGC0000166 | Tylactone\_biosynthetic\_gene\_cluster | 31 | 40.2905740609 | 467 | 1.2e-130 |
| ADM46360.1 | polyketide\_synthase | BGC0000106 | Naphthomycin\_biosynthetic\_gene\_cluster | 31 | 42.5939050319 | 467 | 1.2e-130 |
| ACO94500.1 | polyketide\_synthase\_type\_I | BGC0000097 | ML-449\_biosynthetic\_gene\_cluster | 34 | 35.1169383416 | 467 | 1.2e-130 |
| BAO66528.1 | type\_I\_polyketide\_synthase | BGC0000042 | Cremimycin\_biosynthetic\_gene\_cluster | 33 | 35.2941176471 | 467 | 1.2e-130 |
| BAC68125.1 | modular\_polyketide\_synthase | BGC0000059 | Filipin\_biosynthetic\_gene\_cluster | 32 | 33.1679659816 | 467 | 1.6e-130 |
| AAC46024.1 | polyketide\_synthase\_modules\_1\_and\_2 | BGC0000113 | Niddamycin\_biosynthetic\_gene\_cluster | 32 | 37.98724309 | 467 | 1.6e-130 |
| ADU86004.1 | putative\_modular\_polyketide\_synthase | BGC0000165 | Tiacumicin\_B\_biosynthetic\_gene\_cluster | 32 | 41.3890857548 | 467 | 1.6e-130 |
| ACO94484.1 | polyketide\_synthase\_type\_I | BGC0000097 | ML-449\_biosynthetic\_gene\_cluster | 33 | 35.3649893692 | 467 | 1.6e-130 |
| ADH04658.1 | TugB | BGC0001342 | Thuggacin\_biosynthetic\_gene\_cluster | 33 | 32.7427356485 | 466 | 2e-130 |
| ABP55220.1 | beta-ketoacyl\_synthase | BGC0000142 | Salinilactam\_biosynthetic\_gene\_cluster | 31 | 41.459957477 | 466 | 2e-130 |
| AAO65796.1 | monensin\_polyketide\_synthase\_loading\_module\_and\_module\_1 | BGC0000100 | Monensin\_biosynthetic\_gene\_cluster | 34 | 34.124734231 | 466 | 2e-130 |
| CAA60459.1 | polyketide\_synthase | BGC0001040 | Rapamycin\_biosynthetic\_gene\_cluster | 32 | 41.1764705882 | 466 | 2e-130 |
| AHH99925.1 | PKS\_I | BGC0000002 | Aculeximycin\_biosynthetic\_gene\_cluster | 34 | 32.4946846208 | 466 | 2e-130 |
| AAS98784.1 | JamM | BGC0001001 | Jamaicamide\_biosynthetic\_gene\_cluster | 34 | 32.9907866761 | 466 | 2.7e-130 |
| AKD43768.1 | HerA1 | BGC0001349 | Heronamide\_biosynthetic\_gene\_cluster | 31 | 36.03827073 | 466 | 2.7e-130 |
| AAA79984.2 | soraphen\_polyketide\_synthase\_B | BGC0000147 | Soraphen\_biosynthetic\_gene\_cluster | 33 | 32.8490432318 | 465 | 3.5e-130 |
| AAM81584.2 | putative\_type\_I\_polyketide\_synthase | BGC0000047 | Dihydrochalcomycin\_biosynthetic\_gene\_cluster | 33 | 32.6009922041 | 465 | 3.5e-130 |
| AAZ94387.1 | modular\_polyketide\_synthase | BGC0000040 | Concanamycin\_A\_biosynthetic\_gene\_cluster | 34 | 34.8334514529 | 465 | 4.5e-130 |
| ABC84458.1 | NigAIII | BGC0000114 | Nigericin\_biosynthetic\_gene\_cluster | 32 | 36.428065202 | 465 | 4.5e-130 |
| BAP34739.1 | type\_I\_polyketide\_synthase | BGC0000078 | Incednine\_biosynthetic\_gene\_cluster | 31 | 41.9560595322 | 465 | 4.5e-130 |
| ALA09355.1 | type\_I\_modular\_PKS | BGC0001303 | Lobosamide\_biosynthetic\_gene\_cluster | 33 | 33.0616583983 | 465 | 4.5e-130 |
| AEZ53950.1 | polyketide\_synthase | BGC0000144 | Salinomycin\_biosynthetic\_gene\_cluster | 30 | 42.2749822821 | 465 | 4.5e-130 |
| CAC20919.1 | PimS4\_protein | BGC0000125 | Pimaricin\_biosynthetic\_gene\_cluster | 32 | 36.5343727853 | 465 | 4.5e-130 |
| AEH42474.1 | polyketide\_synthase | BGC0000032 | Calcimycin\_biosynthetic\_gene\_cluster | 32 | 33.4514528703 | 465 | 5.9e-130 |
| AAO65807.1 | monensin\_polyketide\_synthase\_module\_10 | BGC0000100 | Monensin\_biosynthetic\_gene\_cluster | 33 | 33.345145287 | 465 | 5.9e-130 |
| ABB88520.1 | polyketide\_synthase\_type\_I | BGC0000050 | E-837\_biosynthetic\_gene\_cluster | 32 | 36.0028348689 | 465 | 5.9e-130 |
| AKD43764.1 | HerF | BGC0001349 | Heronamide\_biosynthetic\_gene\_cluster | 31 | 41.1055988661 | 464 | 7.7e-130 |
| AAO65799.1 | monensin\_polyketide\_synthase\_modules\_5\_and\_6 | BGC0000100 | Monensin\_biosynthetic\_gene\_cluster | 31 | 40.2905740609 | 464 | 7.7e-130 |
| BAR73017.1 | putative\_PKS\_(KS-AT-KR-ACP-KS-AT-DH-KR-ACP) | BGC0001194 | Hitachimycin\_biosynthetic\_gene\_cluster | 32 | 33.3097094259 | 464 | 7.7e-130 |
| BAK64637.1 | polyketide\_synthase | BGC0000135 | Reveromycin\_biosynthetic\_gene\_cluster | 34 | 32.6009922041 | 464 | 7.7e-130 |
| ABM21569.1 | CrpA | BGC0000975 | Cryptophycin\_biosynthetic\_gene\_cluster | 30 | 44.2948263643 | 464 | 1e-129 |
| AFL48527.1 | laidlomycin\_polyketide\_synthase\_(module\_3\_and\_module\_4) | BGC0000084 | Laidlomycin\_biosynthetic\_gene\_cluster | 31 | 42.1332388377 | 464 | 1e-129 |
| AAQ90173.1 | polyketide\_synthase\_type\_I | BGC0000128 | Pyoluteorin\_biosynthetic\_gene\_cluster | 34 | 32.4592487597 | 464 | 1e-129 |
| CUI25727.1 | Polyketide\_synthase/Nonribosomal\_peptide\_synthase | BGC0001355 | U-68204\_biosynthetic\_gene\_cluster | 31 | 37.6328844791 | 463 | 1.3e-129 |
| ABC84460.1 | NigAV | BGC0000114 | Nigericin\_biosynthetic\_gene\_cluster | 30 | 41.9914953933 | 463 | 1.3e-129 |
| AAO65800.1 | monensin\_polyketide\_synthase\_modules\_7\_and\_8 | BGC0000100 | Monensin\_biosynthetic\_gene\_cluster | 31 | 41.8143160879 | 463 | 1.3e-129 |
| ABK32256.1 | AmbB | BGC0000014 | Ambruticin\_biosynthetic\_gene\_cluster | 32 | 36.3571934798 | 463 | 1.3e-129 |
| AAF71767.1 | NysJ | BGC0000115 | Nystatin\_biosynthetic\_gene\_cluster | 34 | 35.258681786 | 463 | 1.3e-129 |
| AAC38076.1 | polyketide\_synthase\_type\_I | BGC0000127 | Pyoluteorin\_biosynthetic\_gene\_cluster | 34 | 33.5577604536 | 463 | 1.3e-129 |
| CAM00065.1 | EryAIII\_Erythromycin\_polyketide\_synthase\_modules\_5\_and\_6 | BGC0000055 | Erythromycin\_biosynthetic\_gene\_cluster | 33 | 32.7072997874 | 463 | 1.7e-129 |
| AAF86393.1 | FkbB | BGC0000994 | FK520\_biosynthetic\_gene\_cluster | 31 | 42.1686746988 | 463 | 1.7e-129 |
| AFV30249.1 | polyketide\_synthase | BGC0000075 | Hygrocin\_biosynthetic\_gene\_cluster | 32 | 36.3217576187 | 463 | 1.7e-129 |
| ctg1\_orf30 |  | BGC0000096 | Midecamycin\_biosynthetic\_gene\_cluster | 34 | 32.8490432318 | 463 | 2.2e-129 |
| CAJ88187.2 | putative\_type\_I\_modular\_polyketide\_synthase | BGC0000151 | Stambomycin\_biosynthetic\_gene\_cluster | 29 | 40.9638554217 | 462 | 2.9e-129 |
| ADC45515.1 | modular\_polyketide\_synthase | BGC0000093 | Meilingmycin\_biosynthetic\_gene\_cluster | 31 | 41.1764705882 | 462 | 2.9e-129 |
| ACR50773.1 | polyketide\_synthase | BGC0000163 | Tetronasin\_biosynthetic\_gene\_cluster | 31 | 35.3295535082 | 462 | 5e-129 |
| AAF86396.1 | FkbA | BGC0000994 | FK520\_biosynthetic\_gene\_cluster | 30 | 40.4677533664 | 461 | 6.5e-129 |
| ADC45586.1 | modular\_polyketide\_synthase | BGC0000093 | Meilingmycin\_biosynthetic\_gene\_cluster | 33 | 36.5343727853 | 461 | 6.5e-129 |
| ACO94472.1 | polyketide\_synthase\_type\_I | BGC0000029 | BE-14106\_biosynthetic\_gene\_cluster | 32 | 38.1644223955 | 461 | 6.5e-129 |
| ADH04659.1 | TugC | BGC0001342 | Thuggacin\_biosynthetic\_gene\_cluster | 32 | 35.7902197023 | 461 | 6.5e-129 |
| AFL48529.1 | laidlomycin\_polyketide\_synthase\_(module\_5\_and\_module\_6) | BGC0000084 | Laidlomycin\_biosynthetic\_gene\_cluster | 32 | 36.6052445074 | 461 | 8.5e-129 |
| ctg1\_orf253 |  | BGC0001200 | Lorneic\_acid\_A\_biosynthetic\_gene\_cluster | 30 | 41.9206236712 | 460 | 1.5e-128 |
| AAP42857.1 | NanA3 | BGC0000105 | Nanchangmycin\_biosynthetic\_gene\_cluster | 32 | 36.2863217576 | 460 | 1.5e-128 |
| ADU85988.1 | putative\_iterative\_type\_I\_polyketide\_synthase | BGC0000165 | Tiacumicin\_B\_biosynthetic\_gene\_cluster | 33 | 35.6130403969 | 460 | 1.5e-128 |
| AAS79463.1 | polyketide\_synthase\_subunit | BGC0000035 | Chalcomycin\_biosynthetic\_gene\_cluster | 33 | 33.5577604536 | 460 | 1.9e-128 |
| ADX66460.1 | ScnS3 | BGC0000108 | Natamycin\_biosynthetic\_gene\_cluster | 32 | 37.207654146 | 459 | 2.5e-128 |
| ACF35446.1 | MbcAII | BGC0000090 | Macbecin\_biosynthetic\_gene\_cluster | 34 | 32.8136073707 | 459 | 2.5e-128 |
| BAP34733.1 | type\_I\_polyketide\_synthase | BGC0000078 | Incednine\_biosynthetic\_gene\_cluster | 30 | 41.2119064493 | 459 | 2.5e-128 |
| AAU93807.2 | polyketide\_synthase\_modules\_1\_and\_2 | BGC0000054 | Erythromycin\_biosynthetic\_gene\_cluster | 34 | 32.5301204819 | 459 | 2.5e-128 |
| ACB46485.1 | polyketide\_synthase | BGC0000082 | Kijanimicin\_biosynthetic\_gene\_cluster | 32 | 32.8490432318 | 458 | 4.2e-128 |
| CBW54671.1 | polyketide\_synthase/non\_ribosomal\_peptide\_synthetase | BGC0000971 | Cinnabaramide\_biosynthetic\_gene\_cluster | 34 | 32.7072997874 | 458 | 4.2e-128 |
| CAL58683.1 | polyketide\_synthase | BGC0000149 | Spirangien\_biosynthetic\_gene\_cluster | 34 | 33.0262225372 | 458 | 4.2e-128 |
| ABB88533.1 | polyketide\_synthase\_type\_I | BGC0000049 | E-492\_/\_E-975\_biosynthetic\_gene\_cluster | 33 | 33.5577604536 | 458 | 4.2e-128 |
| AFI57005.1 | QmnA1 | BGC0000133 | Quartromicin\_biosynthetic\_gene\_cluster | 34 | 32.3175053154 | 458 | 4.2e-128 |
| ABC87511.1 | polyketide\_synthase | BGC0001011 | Meridamycin\_biosynthetic\_gene\_cluster | 34 | 33.0970942594 | 458 | 5.5e-128 |
| AAZ77673.1 | ChlB1 | BGC0000036 | Chlorothricin\_biosynthetic\_gene\_cluster | 33 | 36.3571934798 | 458 | 5.5e-128 |
| ABC84459.1 | NigAIV | BGC0000114 | Nigericin\_biosynthetic\_gene\_cluster | 31 | 36.7469879518 | 458 | 5.5e-128 |
| ABV83221.1 | CppI | BGC0000116 | Nystatin-like\_Pseudonocardia\_polyene\_biosynthetic\_gene\_cluster | 32 | 32.8844790928 | 458 | 5.5e-128 |
| ctg1\_orf22 |  | BGC0001013 | Meridamycin\_biosynthetic\_gene\_cluster | 34 | 33.0970942594 | 458 | 5.5e-128 |
| ACR50775.1 | polyketide\_synthase | BGC0000163 | Tetronasin\_biosynthetic\_gene\_cluster | 30 | 41.6371367824 | 458 | 7.2e-128 |
| ADU86002.1 | putative\_modular\_polyketide\_synthase | BGC0000165 | Tiacumicin\_B\_biosynthetic\_gene\_cluster | 31 | 40.9992912828 | 457 | 9.4e-128 |
| ACR50785.1 | polyketide\_synthase | BGC0000163 | Tetronasin\_biosynthetic\_gene\_cluster | 32 | 34.5499645641 | 457 | 9.4e-128 |
| ABC87509.1 | polyketide\_synthase | BGC0001011 | Meridamycin\_biosynthetic\_gene\_cluster | 33 | 34.4082211198 | 457 | 1.2e-127 |
| ctg1\_orf20 |  | BGC0001013 | Meridamycin\_biosynthetic\_gene\_cluster | 33 | 34.4082211198 | 457 | 1.2e-127 |
| ACB37743.1 | putative\_type\_I\_polyketide\_synthase | BGC0000162 | Tetrocarcin\_A\_biosynthetic\_gene\_cluster | 33 | 33.9121190645 | 457 | 1.2e-127 |
| CBD77746.1 | non-ribosomal\_peptide\_synthetase/polyketide\_synthase | BGC0000974 | Crocacin\_biosynthetic\_gene\_cluster | 32 | 36.7824238129 | 457 | 1.2e-127 |
| CAE45672.1 | Borrelidin\_polyketide\_synthase,\_type\_I | BGC0000031 | Borrelidin\_biosynthetic\_gene\_cluster | 33 | 33.2034018427 | 457 | 1.2e-127 |
| BAP34734.1 | type\_I\_polyketide\_synthase | BGC0000078 | Incednine\_biosynthetic\_gene\_cluster | 32 | 36.1091424522 | 456 | 1.6e-127 |
| AEK75503.1 | type\_1\_polyketide\_synthase | BGC0000001 | Abyssomicin\_biosynthetic\_gene\_cluster | 33 | 33.4514528703 | 456 | 1.6e-127 |
| AFI57007.1 | QmnA3 | BGC0000133 | Quartromicin\_biosynthetic\_gene\_cluster | 33 | 32.6718639263 | 456 | 1.6e-127 |
| ABX60161.1 | mixed\_NRPS/PKS | BGC0000978 | Cylindrospermopsin\_biosynthetic\_gene\_cluster | 31 | 38.8377037562 | 456 | 2.1e-127 |
| ADF88279.1 | mixed\_NRPS/PKS | BGC0000981 | Cylindrospermopsin\_biosynthetic\_gene\_cluster | 31 | 38.8022678951 | 456 | 2.1e-127 |
| AAF86392.1 | FkbC | BGC0000994 | FK520\_biosynthetic\_gene\_cluster | 30 | 42.6647767541 | 456 | 2.1e-127 |
| CAE02605.1 | polyketide\_synthase\_type\_I | BGC0000024 | Aureothin\_biosynthetic\_gene\_cluster | 31 | 35.3295535082 | 456 | 2.7e-127 |
| AAS79459.1 | polyketide\_synthase\_subunit | BGC0000035 | Chalcomycin\_biosynthetic\_gene\_cluster | 33 | 32.7427356485 | 456 | 2.7e-127 |
| AFL48533.1 | laidlomycin\_polyketide\_synthase\_(module\_10) | BGC0000084 | Laidlomycin\_biosynthetic\_gene\_cluster | 33 | 33.1679659816 | 456 | 2.7e-127 |
| AHE80995.1 | PieA5 | BGC0001169 | Piericidin\_A1\_biosynthetic\_gene\_cluster | 32 | 35.7547838412 | 455 | 3.6e-127 |
| BAO66543.1 | type\_I\_polyketide\_synthase | BGC0000042 | Cremimycin\_biosynthetic\_gene\_cluster | 33 | 32.8136073707 | 455 | 3.6e-127 |
| BAQ25483.1 | type\_I\_polyketide\_synthase | BGC0001288 | Maklamicin\_biosynthetic\_gene\_cluster | 33 | 32.8490432318 | 455 | 3.6e-127 |
| CAJ88177.1 | putative\_type\_I\_polyketide\_synthase | BGC0000151 | Stambomycin\_biosynthetic\_gene\_cluster | 34 | 33.3805811481 | 455 | 4.7e-127 |
| ctg1\_orf9 |  | BGC0000053 | Elaiophylin\_biosynthetic\_gene\_cluster | 33 | 33.2388377038 | 454 | 8e-127 |
| AAC69332.1 | type\_I\_polyketide\_synthase\_PikAIV | BGC0000094 | Methymycin\_/\_pikromycin\_biosynthetic\_gene\_cluster | 32 | 32.3883770376 | 454 | 8e-127 |
| ABB88535.1 | polyketide\_synthase\_type\_I | BGC0000049\_c2 | E-492\_/\_E-975\_biosynthetic\_gene\_cluster | 33 | 33.0616583983 | 454 | 8e-127 |
| BAC76491.1 | lankamycin\_synthase\_LkmAIII | BGC0000085 | Lankamycin\_biosynthetic\_gene\_cluster | 33 | 36.6761162296 | 454 | 1e-126 |
| BAA20102.2 | 6-methylsalicylic\_acid\_synthase | BGC0001276 | 6-methylsalicyclic\_acid\_biosynthetic\_gene\_cluster | 32 | 37.3493975904 | 454 | 1e-126 |
| ALP32045.1 | CycE | BGC0001293 | Cyclizidine\_biosynthetic\_gene\_cluster | 32 | 37.0659107016 | 454 | 1e-126 |
| AJW65410.1 | type\_I\_modular\_polyketide\_synthase | BGC0001195 | Nocardiopsin\_biosynthetic\_gene\_cluster | 33 | 35.2232459249 | 453 | 1.4e-126 |
| AAC68815.1 | FK506\_polyketide\_synthase | BGC0000353 | FK506\_biosynthetic\_gene\_cluster | 32 | 36.428065202 | 453 | 1.8e-126 |
| BAR73021.1 | putative\_PKS\_(KS-AT-KR-ACP) | BGC0001194 | Hitachimycin\_biosynthetic\_gene\_cluster | 33 | 32.8136073707 | 453 | 1.8e-126 |
| AAQ84144.1 | Plm4 | BGC0000123 | Phoslactomycin\_B\_biosynthetic\_gene\_cluster | 34 | 31.3607370659 | 453 | 1.8e-126 |
| CAJ88185.2 | putative\_modular\_polyketide\_synthase | BGC0000151 | Stambomycin\_biosynthetic\_gene\_cluster | 34 | 32.5301204819 | 453 | 1.8e-126 |
| AJO72736.1 | Type\_I\_modular\_polyketide\_synthase | BGC0001381 | Brasilinolide\_biosynthetic\_gene\_cluster | 31 | 36.8532955351 | 452 | 3e-126 |
| ABV99085.1 | thioester\_reductase\_domain | BGC0001007 | Lymphostin\_biosynthetic\_gene\_cluster | 34 | 33.734939759 | 452 | 3e-126 |
| AJW65407.1 | type\_I\_modular\_polyketide\_synthase | BGC0001195 | Nocardiopsin\_biosynthetic\_gene\_cluster | 32 | 35.3295535082 | 452 | 3e-126 |
| AAM54076.1 | polyketide\_synthase | BGC0000020 | Ansamitocin\_biosynthetic\_gene\_cluster | 34 | 33.4160170092 | 452 | 3e-126 |
| BAQ25507.1 | type\_I\_polyketide\_synthase | BGC0001288 | Maklamicin\_biosynthetic\_gene\_cluster | 32 | 35.5421686747 | 451 | 5.2e-126 |
| AEK75502.1 | type\_1\_polyketide\_synthase | BGC0000001 | Abyssomicin\_biosynthetic\_gene\_cluster | 33 | 32.5301204819 | 451 | 5.2e-126 |
| CAQ34918.1 | nonribosomal\_peptide\_synthetase/\_polyketide\_synthase | BGC0000986 | Dkxanthene\_biosynthetic\_gene\_cluster | 29 | 45.1452870305 | 451 | 6.8e-126 |
| AHA38199.1 | GphF | BGC0000069 | Gephyronic\_acid\_biosynthetic\_gene\_cluster | 30 | 43.1254429483 | 451 | 6.8e-126 |
| AAP42855.1 | NanA1 | BGC0000105 | Nanchangmycin\_biosynthetic\_gene\_cluster | 33 | 33.5931963147 | 450 | 1.2e-125 |
| BAG84248.1 | putative\_polyketide\_synthase | BGC0000257 | Prodigiosin\_biosynthetic\_gene\_cluster | 33 | 33.2742735648 | 450 | 1.2e-125 |
| BAC68126.1 | modular\_polyketide\_synthase | BGC0000059 | Filipin\_biosynthetic\_gene\_cluster | 33 | 33.1679659816 | 450 | 1.2e-125 |
| CAA16183.1 | polyketide\_synthase | BGC0001063 | Undecylprodigiosin\_biosynthetic\_gene\_cluster | 34 | 32.1048901488 | 450 | 1.2e-125 |
| EAU32819.1 | 6-methylsalicylic\_acid\_synthase | BGC0000160 | Terreic\_acid\_biosynthetic\_gene\_cluster | 32 | 37.3848334515 | 450 | 1.2e-125 |
| ADC79617.1 | BafAII | BGC0000028 | Bafilomycin\_biosynthetic\_gene\_cluster | 34 | 33.6286321758 | 450 | 1.5e-125 |
| AEH42473.1 | polyketide\_synthase | BGC0000032 | Calcimycin\_biosynthetic\_gene\_cluster | 33 | 32.6718639263 | 450 | 2e-125 |
| CAM00062.1 | EryAI\_Erythromycin\_polyketide\_synthase\_modules\_1\_and\_2 | BGC0000055 | Erythromycin\_biosynthetic\_gene\_cluster | 33 | 33.5223245925 | 450 | 2e-125 |
| CBZ41585.1 | putative\_modular\_polyketide\_synthase | BGC0000151 | Stambomycin\_biosynthetic\_gene\_cluster | 31 | 41.7788802268 | 449 | 2.6e-125 |
| CAJ88175.1 | putative\_polyketide\_synthase\_B | BGC0000151 | Stambomycin\_biosynthetic\_gene\_cluster | 34 | 33.5931963147 | 449 | 3.4e-125 |
| ABB88519.1 | polyketide\_synthase\_type\_I | BGC0000050 | E-837\_biosynthetic\_gene\_cluster | 33 | 34.3019135365 | 448 | 4.4e-125 |
| AHH99922.1 | PKS\_I | BGC0000002 | Aculeximycin\_biosynthetic\_gene\_cluster | 33 | 32.3175053154 | 448 | 4.4e-125 |
| AAQ84146.1 | Plm6 | BGC0000123 | Phoslactomycin\_B\_biosynthetic\_gene\_cluster | 33 | 33.3097094259 | 448 | 5.7e-125 |
| ABY66019.1 | 6-methylsalicylic\_acid\_synthase | BGC0001008 | Maduropeptin\_biosynthetic\_gene\_cluster | 32 | 36.6052445074 | 448 | 7.5e-125 |
| AAF71774.1 | NysA | BGC0000115 | Nystatin\_biosynthetic\_gene\_cluster | 32 | 35.0460666194 | 447 | 9.8e-125 |
| AEZ53947.1 | polyketide\_synthase | BGC0000144 | Salinomycin\_biosynthetic\_gene\_cluster | 33 | 33.0262225372 | 447 | 9.8e-125 |
| AAQ90174.1 | polyketide\_synthase\_type\_I | BGC0000128 | Pyoluteorin\_biosynthetic\_gene\_cluster | 32 | 33.1679659816 | 447 | 9.8e-125 |
| BAE93728.1 | type\_I\_polyketide\_synthase | BGC0000164 | Tetronomycin\_biosynthetic\_gene\_cluster | 33 | 32.8490432318 | 446 | 1.7e-124 |
| ALP32046.1 | CycF | BGC0001293 | Cyclizidine\_biosynthetic\_gene\_cluster | 30 | 41.7080085046 | 446 | 1.7e-124 |
| CCP20049.1 | divL2\_protein | BGC0001119 | Divergolide\_biosynthetic\_gene\_cluster | 31 | 35.7902197023 | 446 | 1.7e-124 |
| ctg1\_orf8 |  | BGC0000053 | Elaiophylin\_biosynthetic\_gene\_cluster | 32 | 33.2388377038 | 446 | 2.2e-124 |
| CAI94682.1 | putative\_polyketide\_synthase | BGC0000141 | Rubradirin\_biosynthetic\_gene\_cluster | 33 | 33.0970942594 | 446 | 2.2e-124 |
| ACR50791.1 | putative\_polyketide\_synthase | BGC0000163 | Tetronasin\_biosynthetic\_gene\_cluster | 29 | 42.3812898653 | 446 | 2.8e-124 |
| AAC46027.1 | polyketide\_synthase\_module\_6 | BGC0000113 | Niddamycin\_biosynthetic\_gene\_cluster | 33 | 32.8490432318 | 446 | 2.8e-124 |
| ABP73645.1 | SalA | BGC0000145 | Salinosporamide\_A\_biosynthetic\_gene\_cluster | 34 | 31.6442239546 | 446 | 2.8e-124 |
| BI06\_RS39060 | polyketide\_synthase | BGC0001232 | Leucanicidin\_biosynthetic\_gene\_cluster | 33 | 33.4160170092 | 445 | 3.7e-124 |
| ctg1\_orf523 |  | BGC0001199 | Akaeolide\_biosynthetic\_gene\_cluster | 33 | 32.5301204819 | 445 | 3.7e-124 |
| AAK83194.1 | polyketide\_synthase | BGC0000026 | Avilamycin\_A\_biosynthetic\_gene\_cluster | 32 | 35.7547838412 | 445 | 3.7e-124 |
| ANC94962.1 | AlmHV | BGC0001396 | Aldgamycin\_biosynthetic\_gene\_cluster | 32 | 33.5577604536 | 445 | 3.7e-124 |
| BAF02925.1 | type\_I\_polyketide\_synthase | BGC0000073 | Halstoctacosanolide\_biosynthetic\_gene\_cluster | 31 | 33.5223245925 | 445 | 4.8e-124 |
| ABP55493.1 | thioester\_reductase\_domain | BGC0001006 | Lymphostin\_biosynthetic\_gene\_cluster | 34 | 32.6718639263 | 445 | 4.8e-124 |
| ABP53498.1 | beta-ketoacyl\_synthase | BGC0001041 | Salinosporamide\_biosynthetic\_gene\_cluster | 34 | 31.6442239546 | 445 | 6.3e-124 |
| AFU82614.1 | mixed\_NRPS\_PKS | BGC0000998 | Guadinomine\_biosynthetic\_gene\_cluster | 31 | 37.0659107016 | 445 | 6.3e-124 |
| ABB52545.1 | putative\_type\_I\_polyketide\_synthase | BGC0000047 | Dihydrochalcomycin\_biosynthetic\_gene\_cluster | 32 | 33.5577604536 | 443 | 1.4e-123 |
| ACF35447.1 | MbcAIII | BGC0000090 | Macbecin\_biosynthetic\_gene\_cluster | 31 | 36.5698086464 | 443 | 1.4e-123 |
| CAA60460.1 | polyketide\_synthase | BGC0001040 | Rapamycin\_biosynthetic\_gene\_cluster | 29 | 41.8143160879 | 443 | 1.8e-123 |
| ABO15861.1 | polyketide\_synthase | BGC0000130 | Pyrrolomycin\_biosynthetic\_gene\_cluster | 33 | 33.6286321758 | 442 | 3.1e-123 |
| ADC79619.1 | BafAIV | BGC0000028 | Bafilomycin\_biosynthetic\_gene\_cluster | 33 | 34.479092842 | 442 | 4.1e-123 |
| AAT70102.1 | CurG | BGC0001165 | Curacin\_biosynthetic\_gene\_cluster | 32 | 33.2742735648 | 442 | 4.1e-123 |
| AEE88283.1 | CurG | BGC0000976 | Curacin\_biosynthetic\_gene\_cluster | 32 | 33.2742735648 | 442 | 4.1e-123 |
| AAC01713.1 | RifD | BGC0000136 | Rifamycin\_biosynthetic\_gene\_cluster | 31 | 36.2863217576 | 441 | 5.4e-123 |
| ADC45534.1 | modular\_polyketide\_synthase | BGC0000093 | Meilingmycin\_biosynthetic\_gene\_cluster | 33 | 33.2034018427 | 441 | 5.4e-123 |
| ACO94488.1 | polyketide\_synthase\_type\_I | BGC0000097 | ML-449\_biosynthetic\_gene\_cluster | 29 | 41.2827781715 | 441 | 9.1e-123 |
| ABP55222.1 | beta-ketoacyl\_synthase | BGC0000142 | Salinilactam\_biosynthetic\_gene\_cluster | 32 | 33.1679659816 | 441 | 9.1e-123 |
| AHH99923.1 | PKS\_I | BGC0000002 | Aculeximycin\_biosynthetic\_gene\_cluster | 30 | 45.0389794472 | 440 | 1.2e-122 |
| AAB66508.1 | tylactone\_synthase\_module\_7 | BGC0000166 | Tylactone\_biosynthetic\_gene\_cluster | 33 | 32.955350815 | 440 | 1.2e-122 |
| ABK32259.1 | AmbE | BGC0000014 | Ambruticin\_biosynthetic\_gene\_cluster | 29 | 42.7002126152 | 440 | 1.2e-122 |
| ABY21538.1 | AngAI | BGC0000018 | Angolamycin\_biosynthetic\_gene\_cluster | 33 | 32.4238128987 | 440 | 1.6e-122 |
| ABY83164.1 | Azi26 | BGC0000960 | Azinomycin\_B\_biosynthetic\_gene\_cluster | 30 | 37.2785258682 | 440 | 2e-122 |
| AHH99924.1 | PKS\_I | BGC0000002 | Aculeximycin\_biosynthetic\_gene\_cluster | 31 | 39.6172927002 | 439 | 2.7e-122 |
| CAA60462.1 | polyketide\_synthase | BGC0001040 | Rapamycin\_biosynthetic\_gene\_cluster | 32 | 35.7902197023 | 439 | 2.7e-122 |
| ADC79616.1 | BafAI | BGC0000028 | Bafilomycin\_biosynthetic\_gene\_cluster | 32 | 32.7427356485 | 438 | 4.5e-122 |
| ACA99172.1 | Polyketide\_synthase | BGC0001160 | Nonadecene\_biosynthetic\_gene\_cluster | 33 | 32.3883770376 | 438 | 4.5e-122 |
| ABY21540.1 | AngAIII | BGC0000018 | Angolamycin\_biosynthetic\_gene\_cluster | 31 | 37.1722182849 | 438 | 7.7e-122 |
| EYT83439.1 | beta-ketoacyl\_synthase | BGC0001213 | Nataxazole\_biosynthetic\_gene\_cluster | 33 | 34.124734231 | 437 | 1e-121 |
| AAP42860.1 | NanA6 | BGC0000105 | Nanchangmycin\_biosynthetic\_gene\_cluster | 31 | 34.6208362863 | 437 | 1.3e-121 |
| AAC69331.1 | type\_I\_polyketide\_synthase\_PikAIII | BGC0000094 | Methymycin\_/\_pikromycin\_biosynthetic\_gene\_cluster | 31 | 33.6995038979 | 437 | 1.3e-121 |
| CAQ18839.1 | hybrid\_polyketide\_synthase/nonribosomal\_polypetide\_synthetase | BGC0000954 | Ajudazol\_biosynthetic\_gene\_cluster | 33 | 34.0538625089 | 437 | 1.3e-121 |
| AAG13919.1 | megalomicin\_6-deoxyerythronolide\_B\_synthase\_3 | BGC0000092 | Megalomicin\_biosynthetic\_gene\_cluster | 33 | 32.955350815 | 436 | 1.7e-121 |
| AJO72737.1 | Type\_I\_modular\_polyketide\_synthase | BGC0001381 | Brasilinolide\_biosynthetic\_gene\_cluster | 32 | 33.3097094259 | 436 | 2.9e-121 |
| BAE93722.1 | type\_I\_polyketide\_synthase | BGC0000164 | Tetronomycin\_biosynthetic\_gene\_cluster | 33 | 33.3805811481 | 436 | 2.9e-121 |
| BI06\_RS39075 | polyketide\_synthase | BGC0001232 | Leucanicidin\_biosynthetic\_gene\_cluster | 33 | 33.4868887314 | 436 | 2.9e-121 |
| ALJ49910.1 | TlmH | BGC0001237 | Thiolactomycin\_biosynthetic\_gene\_cluster | 30 | 41.8851878101 | 435 | 3.8e-121 |
| ABI91470.1 | beta-ketoacyl\_synthase | BGC0001094 | Enacyloxin\_biosynthetic\_gene\_cluster | 31 | 36.3571934798 | 435 | 5e-121 |
| AIG62146.1 | 6-methylsalicylic\_acid\_synthase | BGC0000120 | Patulin\_biosynthetic\_gene\_cluster | 31 | 37.2785258682 | 435 | 5e-121 |
| AAF71768.1 | NysK | BGC0000115 | Nystatin\_biosynthetic\_gene\_cluster | 32 | 35.2232459249 | 435 | 6.6e-121 |
| ABO15888.1 | polyketide\_synthase | BGC0000132 | Pyrrolomycin\_biosynthetic\_gene\_cluster | 35 | 27.533664068 | 434 | 8.6e-121 |
| AAM54078.1 | polyketide\_synthase | BGC0000020 | Ansamitocin\_biosynthetic\_gene\_cluster | 31 | 35.8965272856 | 434 | 8.6e-121 |
| AAS98200.1 | MSAS-type\_polyketide\_synthase | BGC0001273 | Asperlactone\_biosynthetic\_gene\_cluster | 32 | 36.2154500354 | 434 | 1.1e-120 |
| IF55\_RS36525 | polyketide\_synthase | BGC0001348 | JBIR-100\_biosynthetic\_gene\_cluster | 33 | 33.2388377038 | 433 | 1.5e-120 |
| AHA38202.1 | GphI | BGC0000069 | Gephyronic\_acid\_biosynthetic\_gene\_cluster | 32 | 35.8610914245 | 433 | 1.9e-120 |
| ABY21541.1 | AngAIV | BGC0000018 | Angolamycin\_biosynthetic\_gene\_cluster | 32 | 34.08929837 | 433 | 1.9e-120 |
| ctg1\_orf524 |  | BGC0001199 | Akaeolide\_biosynthetic\_gene\_cluster | 33 | 32.6718639263 | 433 | 1.9e-120 |
| CBZ41586.1 | putative\_modular\_polyketide\_synthase | BGC0000151 | Stambomycin\_biosynthetic\_gene\_cluster | 33 | 32.4238128987 | 432 | 3.3e-120 |
| CCP20051.1 | divM\_protein | BGC0001119 | Divergolide\_biosynthetic\_gene\_cluster | 32 | 32.6718639263 | 432 | 3.3e-120 |
| AAP42856.1 | NanA2 | BGC0000105 | Nanchangmycin\_biosynthetic\_gene\_cluster | 32 | 32.955350815 | 432 | 4.2e-120 |
| AAM54077.1 | polyketide\_synthase | BGC0000020 | Ansamitocin\_biosynthetic\_gene\_cluster | 34 | 32.6364280652 | 431 | 5.5e-120 |
| AAW03327.1 | CtaD | BGC0000982 | Cystothiazole\_A\_biosynthetic\_gene\_cluster | 28 | 42.4167257264 | 431 | 7.2e-120 |
| AHD05619.1 | putative\_polyketide\_synthase\_subunit | BGC0001033 | Paenilamicin\_biosynthetic\_gene\_cluster | 32 | 33.2034018427 | 430 | 1.2e-119 |
| BAF85839.1 | modular\_polyketide\_synthase | BGC0000109 | Nemadectin\_biosynthetic\_gene\_cluster | 32 | 33.4514528703 | 430 | 2.1e-119 |
| AFP87524.1 | type\_I\_polyketide\_synthase | BGC0001159 | Marinopyrrole\_biosynthetic\_gene\_cluster | 33 | 31.6087880936 | 428 | 4.7e-119 |
| AAP85336.1 | type\_I\_PKS | BGC0000233 | Hedamycin\_biosynthetic\_gene\_cluster | 33 | 32.9199149539 | 428 | 6.1e-119 |
| AAF00957.1 | McyG | BGC0001017 | Microcystin\_biosynthetic\_gene\_cluster | 31 | 33.5931963147 | 428 | 6.1e-119 |
| BAP34763.1 | type\_I\_polyketide\_synthase | BGC0000078 | Incednine\_biosynthetic\_gene\_cluster | 32 | 35.9319631467 | 428 | 8e-119 |
| AAM77986.1 | iterative\_type\_I\_polyketide\_synthase | BGC0000112 | Neocarzinostatin\_biosynthetic\_gene\_cluster | 31 | 36.428065202 | 427 | 1e-118 |
| AEZ53948.1 | polyketide\_synthase | BGC0000144 | Salinomycin\_biosynthetic\_gene\_cluster | 32 | 32.9199149539 | 427 | 1e-118 |
| AEC13072.1 | fosF | BGC0000060 | Fostriecin\_biosynthetic\_gene\_cluster | 31 | 33.4514528703 | 427 | 1.4e-118 |
| AHF22854.1 | MarL | BGC0000091 | Marineosin\_biosynthetic\_gene\_cluster | 33 | 33.3805811481 | 426 | 1.8e-118 |
| ABG02264.1 | SalB | BGC0000143 | Salinomycin\_biosynthetic\_gene\_cluster | 33 | 33.0262225372 | 425 | 5.2e-118 |
| AEC13079.1 | fosA | BGC0000060 | Fostriecin\_biosynthetic\_gene\_cluster | 37 | 25.9390503189 | 425 | 6.8e-118 |
| AHB82057.1 | polyketide\_synthase | BGC0001019 | Microsclerodermins\_biosynthetic\_gene\_cluster | 33 | 32.5301204819 | 424 | 8.9e-118 |
| CAD29795.1 | peptide\_synthetase | BGC0001015 | Microcystin\_biosynthetic\_gene\_cluster | 31 | 33.2742735648 | 424 | 8.9e-118 |
| CAD15508.1 | probable\_polyketide\_synthase\_protein | BGC0001014 | Micacocidin\_biosynthetic\_gene\_cluster | 33 | 32.955350815 | 423 | 2e-117 |
| AAS98787.1 | JamP | BGC0001001 | Jamaicamide\_biosynthetic\_gene\_cluster | 34 | 32.955350815 | 423 | 2.6e-117 |
| CCA29203.1 | non-ribosomal\_peptide\_synthetase/polyketide\_synthase | BGC0000955 | Althiomycin\_biosynthetic\_gene\_cluster | 33 | 33.6286321758 | 422 | 3.4e-117 |
| CBA11582.1 | polyketide\_synthase\_type\_I | BGC0001046 | Streptolydigin\_biosynthetic\_gene\_cluster | 31 | 36.2508858965 | 422 | 4.4e-117 |
| ABV83222.1 | CppJ | BGC0000116 | Nystatin-like\_Pseudonocardia\_polyene\_biosynthetic\_gene\_cluster | 32 | 35.5067328136 | 420 | 1.3e-116 |
| AHB82062.1 | polyketide\_synthase | BGC0001231 | Microsclerodermins\_biosynthetic\_gene\_cluster | 28 | 46.137491141 | 420 | 1.3e-116 |
| AJO72743.1 | Type\_I\_modular\_polyketide\_synthase | BGC0001381 | Brasilinolide\_biosynthetic\_gene\_cluster | 35 | 32.5301204819 | 418 | 8.3e-116 |
| CAE14173.1 |  | BGC0000383 | Luminmycin\_biosynthetic\_gene\_cluster | 31 | 36.428065202 | 417 | 1.1e-115 |
| ADZ24995.1 | non-ribosomal\_peptide\_synthase/polyketide\_synthase | BGC0000380 | Leupyrrin\_biosynthetic\_gene\_cluster | 28 | 43.0545712261 | 417 | 1.4e-115 |
| AAX35547.1 | polyketide\_syntase\_2 | BGC0001275 | 6-methylsalicyclic\_acid\_biosynthetic\_gene\_cluster | 31 | 35.8610914245 | 417 | 1.4e-115 |
| AJO72734.1 | Type\_I\_modular\_polyketide\_synthase | BGC0001381 | Brasilinolide\_biosynthetic\_gene\_cluster | 32 | 33.8412473423 | 416 | 1.8e-115 |
| CAD89775.1 | MelD\_protein | BGC0001009 | Melithiazol\_biosynthetic\_gene\_cluster | 28 | 42.8065201984 | 416 | 1.8e-115 |
| AHB82065.1 | polyketide\_synthase | BGC0001231 | Microsclerodermins\_biosynthetic\_gene\_cluster | 38 | 23.5294117647 | 416 | 2.4e-115 |
| AHA38201.1 | GphH | BGC0000069 | Gephyronic\_acid\_biosynthetic\_gene\_cluster | 34 | 28.7384833451 | 415 | 4.1e-115 |
| ABY21542.1 | AngAV | BGC0000018 | Angolamycin\_biosynthetic\_gene\_cluster | 31 | 33.9829907867 | 415 | 5.4e-115 |
| AHB82070.1 | polyketide\_synthase | BGC0001231 | Microsclerodermins\_biosynthetic\_gene\_cluster | 32 | 32.7427356485 | 415 | 7e-115 |
| AAD03048.1 | type\_I\_polyketide\_synthase | BGC0000041 | Coronafacic\_acid\_biosynthetic\_gene\_cluster | 31 | 35.8965272856 | 414 | 9.2e-115 |
| AGY62753.1 | EbeA | BGC0000051 | Ebelactone\_biosynthetic\_gene\_cluster | 33 | 29.8724309001 | 414 | 9.2e-115 |
| CAQ34929.1 | putative\_polyketide\_synthase | BGC0000986 | Dkxanthene\_biosynthetic\_gene\_cluster | 32 | 32.8136073707 | 414 | 1.2e-114 |
| AGZ15472.1 | putative\_modular\_polyketide\_synthase | BGC0001036 | Polyoxypeptin\_biosynthetic\_gene\_cluster | 33 | 32.6009922041 | 413 | 2.7e-114 |
| AGC24271.1 | PrlQ | BGC0001038 | Pyralomicin\_biosynthetic\_gene\_cluster | 33 | 29.8724309001 | 412 | 4.5e-114 |
| AAO62585.1 | peptide\_sythetase\_polyketide\_synthase\_fusion\_protein | BGC0001016 | Microcystin\_biosynthetic\_gene\_cluster | 31 | 33.6640680369 | 410 | 1.3e-113 |
| ABI91466.1 | beta-ketoacyl\_synthase | BGC0001094 | Enacyloxin\_biosynthetic\_gene\_cluster | 32 | 32.6364280652 | 409 | 3.8e-113 |
| AJO72735.1 | Type\_I\_modular\_polyketide\_synthase | BGC0001381 | Brasilinolide\_biosynthetic\_gene\_cluster | 29 | 45.357902197 | 409 | 3.8e-113 |
| AHB82059.1 | non\_ribosomal\_peptide\_synthetase/polyketide\_synthase | BGC0001019 | Microsclerodermins\_biosynthetic\_gene\_cluster | 32 | 33.9475549256 | 408 | 5e-113 |
| AHH99920.1 | PKS\_I | BGC0000002 | Aculeximycin\_biosynthetic\_gene\_cluster | 32 | 32.6718639263 | 407 | 1.1e-112 |
| AHD05614.1 | putative\_non-ribosomal\_peptide\_ligase/\_polyketide\_synthase\_hybrid | BGC0001033 | Paenilamicin\_biosynthetic\_gene\_cluster | 31 | 32.955350815 | 407 | 1.5e-112 |
| ADC45516.1 | modular\_polyketide\_synthase | BGC0000093 | Meilingmycin\_biosynthetic\_gene\_cluster | 33 | 32.565556343 | 406 | 2.5e-112 |
| AIW00670.1 | mellein\_synthase | BGC0001244 | (-)-Mellein\_biosynthetic\_gene\_cluster | 30 | 38.3416017009 | 406 | 3.3e-112 |
| AHA38200.1 | GphG | BGC0000069 | Gephyronic\_acid\_biosynthetic\_gene\_cluster | 34 | 29.9078667612 | 405 | 4.3e-112 |
| AHB82054.1 | polyketide\_synthase | BGC0001019 | Microsclerodermins\_biosynthetic\_gene\_cluster | 38 | 23.7065910702 | 405 | 4.3e-112 |
| CAL80821.1 | sylD-like\_NRPS/PKS | BGC0000997 | Glidobactin\_biosynthetic\_gene\_cluster | 33 | 34.1601700921 | 405 | 5.6e-112 |
| ACZ65476.1 | type\_I\_modular\_polyketide\_synthase | BGC0000140 | RK-682\_biosynthetic\_gene\_cluster | 33 | 29.8015591779 | 404 | 1.2e-111 |
| EPE34340.1 | Thiolase-like\_protein | BGC0001035 | Pneumocandin\_biosynthetic\_gene\_cluster | 32 | 31.7505315379 | 403 | 1.6e-111 |
| AAP42858.1 | NanA4 | BGC0000105 | Nanchangmycin\_biosynthetic\_gene\_cluster | 29 | 37.5620127569 | 403 | 2.1e-111 |
| AJO72742.1 | Type\_I\_modular\_polyketide\_synthase | BGC0001381 | Brasilinolide\_biosynthetic\_gene\_cluster | 34 | 29.6598157335 | 403 | 2.1e-111 |
| AAR87760.2 | ZmaK | BGC0001059 | Zwittermycin\_A\_biosynthetic\_gene\_cluster | 32 | 32.7427356485 | 402 | 3.6e-111 |
| OAQ83765.1 | KR\_domain-containing\_protein | BGC0001358 | Leucinostatins\_biosynthetic\_gene\_cluster | 32 | 32.4946846208 | 399 | 4e-110 |
| OAQ83760.1 | polyketide\_synthase | BGC0001358 | Leucinostatins\_biosynthetic\_gene\_cluster | 32 | 31.3607370659 | 398 | 5.2e-110 |
| ABF87031.1 | non-ribosomal\_peptide\_synthetase/polyketide\_synthase | BGC0000393 | Myxoprincomide\_biosynthetic\_gene\_cluster | 30 | 33.2034018427 | 397 | 1.2e-109 |
| EJK79843.1 | amino\_acid\_adenylation\_enzyme/thioester\_reductase\_family\_protein | BGC0000436 | Syringolin\_A\_biosynthetic\_gene\_cluster | 31 | 36.3571934798 | 396 | 2e-109 |
| AHB82063.1 | polyketide\_synthase | BGC0001231 | Microsclerodermins\_biosynthetic\_gene\_cluster | 37 | 25.6201275691 | 396 | 2.6e-109 |
| AHB82072.1 | non\_ribosomal\_peptide\_synthetase/polyketide\_synthase | BGC0001231 | Microsclerodermins\_biosynthetic\_gene\_cluster | 32 | 33.4514528703 | 394 | 7.5e-109 |
| CAJ76291.1 | putative\_polyketide\_synthase | BGC0000972 | Colibactin\_biosynthetic\_gene\_cluster | 31 | 32.9907866761 | 394 | 9.8e-109 |
| BAE93725.1 | type\_I\_polyketide\_synthase | BGC0000164 | Tetronomycin\_biosynthetic\_gene\_cluster | 31 | 33.6286321758 | 394 | 9.8e-109 |
| CAF05649.1 | TubD\_protein | BGC0001053 | Tubulysin\_biosynthetic\_gene\_cluster | 32 | 33.8058114812 | 394 | 9.8e-109 |
| ACB12550.1 | Fum1 | BGC0000063 | Fumonisin\_biosynthetic\_gene\_cluster | 31 | 33.5931963147 | 394 | 9.8e-109 |
| BAQ25482.1 | type\_I\_polyketide\_synthase | BGC0001288 | Maklamicin\_biosynthetic\_gene\_cluster | 37 | 23.3876683203 | 394 | 1.3e-108 |
| ACM79805.1 | ZmaA | BGC0001059 | Zwittermycin\_A\_biosynthetic\_gene\_cluster | 31 | 29.5535081502 | 393 | 2.2e-108 |
| AEF33079.1 | polyketide\_synthase | BGC0001039 | Pyridomycin\_biosynthetic\_gene\_cluster | 31 | 33.2388377038 | 393 | 2.2e-108 |
| EHA22196.1 | polyketide\_synthase | BGC0000170 | Yanuthone\_D\_biosynthetic\_gene\_cluster | 30 | 35.5067328136 | 392 | 3.7e-108 |
| ADA69241.1 | cis-AT\_polyketide\_synthase | BGC0001071 | Nosperin\_biosynthetic\_gene\_cluster | 31 | 32.8136073707 | 391 | 1.1e-107 |
| AHD05615.1 | putative\_non-ribosomal\_peptide\_ligase/\_polyketide\_synthase\_hybrid | BGC0001033 | Paenilamicin\_biosynthetic\_gene\_cluster | 30 | 32.7781715096 | 390 | 1.4e-107 |
| AAF15892.2 | NosB | BGC0001028 | Nostopeptolide\_biosynthetic\_gene\_cluster | 32 | 33.0970942594 | 390 | 1.9e-107 |
| AHB82052.1 | polyketide\_synthase | BGC0001019 | Microsclerodermins\_biosynthetic\_gene\_cluster | 37 | 23.5648476258 | 388 | 9.2e-107 |
| AEK75504.1 | type\_1\_polyketide\_synthase | BGC0000001 | Abyssomicin\_biosynthetic\_gene\_cluster | 31 | 32.565556343 | 386 | 3.5e-106 |
| CAO98848.1 | polyketide\_synthase\_AufE | BGC0000023 | Aurafuron\_biosynthetic\_gene\_cluster | 36 | 23.7774627923 | 384 | 7.8e-106 |
| AFV52200.1 | polyketide\_synthase\_module | BGC0000081 | Kedarcidin\_biosynthetic\_gene\_cluster | 32 | 33.2034018427 | 384 | 1.3e-105 |
| BAQ25511.1 | type\_I\_polyketide\_synthase | BGC0001288 | Maklamicin\_biosynthetic\_gene\_cluster | 37 | 24.7342310418 | 381 | 6.6e-105 |
| CAF05651.1 | TubF\_protein | BGC0001053 | Tubulysin\_biosynthetic\_gene\_cluster | 31 | 33.0970942594 | 380 | 1.9e-104 |
| ADF88265.1 | mixed\_nonribosomal\_peptide\_synthetase/\_polyketide\_synthase | BGC0000980 | Cylindrospermopsin\_biosynthetic\_gene\_cluster | 34 | 26.0453579022 | 380 | 1.9e-104 |
| ADF88262.1 | mixed\_nonribosomal\_peptide\_synthetase/\_polyketide\_synthase | BGC0000979 | Cylindrospermopsin\_biosynthetic\_gene\_cluster | 34 | 26.0453579022 | 380 | 1.9e-104 |
| EFY94490.1 | polyketide\_synthase,\_putative | BGC0000337 | Destruxins\_biosynthetic\_gene\_cluster | 29 | 39.2629340893 | 379 | 2.5e-104 |
| AAD43562.2 | Fum1p | BGC0000062 | Fumonisin\_biosynthetic\_gene\_cluster | 31 | 31.5379163714 | 379 | 4.3e-104 |
| BAJ09789.1 | polyketide\_synthase | BGC0000146 | Solanapyrone\_biosynthetic\_gene\_cluster | 35 | 27.6399716513 | 376 | 3.6e-103 |
| CAD70195.1 | non-ribosomal\_peptide\_synthetase | BGC0001047 | Syringolin\_A\_biosynthetic\_gene\_cluster | 33 | 29.9787384833 | 376 | 3.6e-103 |
| CAQ34917.1 | polyketide\_synthase | BGC0000986 | Dkxanthene\_biosynthetic\_gene\_cluster | 35 | 23.3876683203 | 376 | 3.6e-103 |
| AFD30954.1 | CrmA | BGC0000966 | Caerulomycin\_A\_biosynthetic\_gene\_cluster | 31 | 33.4160170092 | 374 | 8e-103 |
| ADH04682.1 | polyketide\_synthase | BGC0001344 | Tubulysin\_biosynthetic\_gene\_cluster | 31 | 35.5067328136 | 373 | 3.1e-102 |
| ABL74938.1 | TlmVIII | BGC0001048 | Tallysomycin\_biosynthetic\_gene\_cluster | 31 | 33.5223245925 | 371 | 8.9e-102 |
| AKA54627.1 | PKS | BGC0001216 | Splenocin\_biosynthetic\_gene\_cluster | 30 | 32.9907866761 | 369 | 2.6e-101 |
| CBF74114.1 | Conidial\_yellow\_pigment\_biosynthesis\_polyketide\_synthase\_(PKS)(EC\_2.3.1.-)\_[Source:UniProtKB/Swiss-Prot;Acc:Q03149] | BGC0000107 | Naphthopyrone\_biosynthetic\_gene\_cluster | 30 | 33.8412473423 | 369 | 3.4e-101 |
| WP\_010639241.1 | beta-ketoacyl\_synthase | BGC0000958 | Antimycin\_biosynthetic\_gene\_cluster | 31 | 32.9199149539 | 368 | 5.8e-101 |
| ADH04680.1 | hybrid\_polyketide\_synthase/non-ribosomal\_peptide\_synthetase | BGC0001344 | Tubulysin\_biosynthetic\_gene\_cluster | 31 | 33.5931963147 | 367 | 1.7e-100 |
| EAL84397.1 | polyketide\_synthase,\_putative | BGC0001118 | Endocrocin\_biosynthetic\_gene\_cluster | 30 | 32.9907866761 | 361 | 1.2e-98 |
| AAP33839.2 | ochratoxin\_A\_polyketide\_synthase | BGC0001030 | Ochratoxin\_A\_biosynthetic\_gene\_cluster | 33 | 26.6832034018 | 356 | 3e-97 |
| ACD39770.1 | non-reducing\_polyketide\_synthase | BGC0000134 | Radicicol\_biosynthetic\_gene\_cluster | 29 | 33.2034018427 | 355 | 5.1e-97 |
| AEP40925.1 | polyketide\_synthase\_type\_I | BGC0000021 | Apoptolidin\_biosynthetic\_gene\_cluster | 28 | 41.2119064493 | 353 | 1.9e-96 |
| ACR50795.1 | putative\_polyketide\_synthase | BGC0000163 | Tetronasin\_biosynthetic\_gene\_cluster | 33 | 23.0687455705 | 353 | 2.5e-96 |
| EAU38791.1 | hypothetical\_protein | BGC0000161 | Terrein\_biosynthetic\_gene\_cluster | 28 | 37.5265768958 | 353 | 2.5e-96 |
| AAZ95017.1 | polyketide\_synthase | BGC0000048 | Dothistromin\_biosynthetic\_gene\_cluster | 30 | 32.7781715096 | 352 | 4.3e-96 |
| ABX37384.1 | Beta-ketoacyl\_synthase | BGC0000984 | Delftibactin\_biosynthetic\_gene\_cluster | 29 | 33.5577604536 | 352 | 5.6e-96 |
| ABB90282.1 | polyketide\_synthase | BGC0001057 | Zearalenone\_biosynthetic\_gene\_cluster | 28 | 39.6881644224 | 351 | 7.3e-96 |
| ADA82585.1 | hybrid\_trans-AT\_polyketide\_synthase\_-\_nonribosomal\_peptide\_synthetase | BGC0001110 | Psymberin\_biosynthetic\_gene\_cluster | 35 | 23.2104890149 | 351 | 9.5e-96 |
| AGY62756.1 | EbeD | BGC0000051 | Ebelactone\_biosynthetic\_gene\_cluster | 35 | 21.6513111269 | 350 | 1.6e-95 |
| AGC95321.1 | CurS2 | BGC0000045 | Dehydrocurvularin\_biosynthetic\_gene\_cluster | 28 | 33.0262225372 | 348 | 6.2e-95 |
| EGD99348.1 | polyketide\_synthase | BGC0001144 | Neosartoricin\_biosynthetic\_gene\_cluster | 30 | 33.9475549256 | 348 | 8.1e-95 |
| ADM79459.1 | PKS16\_protein | BGC0001266 | Grayanic\_acid\_biosynthetic\_gene\_cluster | 29 | 33.5223245925 | 346 | 2.3e-94 |
| AEZ54376.1 | PieA3 | BGC0000124 | Piericidin\_A1\_biosynthetic\_gene\_cluster | 34 | 23.4939759036 | 346 | 4e-94 |
| CAQ43079.1 | polyketide\_synthase | BGC0000970 | Chondrochloren\_biosynthetic\_gene\_cluster | 37 | 20.4819277108 | 345 | 6.8e-94 |
| ABU63483.1 | polyketide\_synthase | BGC0001272 | Elsinochrome\_B\_/\_elsinochrome\_C\_biosynthetic\_gene\_cluster | 28 | 33.6640680369 | 344 | 1.2e-93 |
| CCT67991.1 | bikaverin\_cluster-polyketide\_synthase | BGC0000030 | Bikaverin\_biosynthetic\_gene\_cluster | 29 | 33.0970942594 | 343 | 2.6e-93 |
| ACD39762.1 | non-reducing\_polyketide\_synthase | BGC0000077 | Hypothemycin\_biosynthetic\_gene\_cluster | 29 | 32.6009922041 | 341 | 9.9e-93 |
| ACD39753.1 | non-reducing\_polyketide\_synthase | BGC0000076 | Hypothemycin\_biosynthetic\_gene\_cluster | 29 | 32.6009922041 | 341 | 9.9e-93 |
| CCH26295.1 | putative\_polyketide\_synthase | BGC0001158\_c6 | Bikaverin\_biosynthetic\_gene\_cluster | 28 | 32.9199149539 | 341 | 9.9e-93 |
| ADI59531.1 | CorI | BGC0001091 | Corallopyronin\_biosynthetic\_gene\_cluster | 36 | 20.163004961 | 340 | 2.2e-92 |
| ACR33077.1 | polyketide\_synthase | BGC0000017 | Anatoxin\_biosynthetic\_gene\_cluster | 30 | 31.0772501772 | 337 | 1.9e-91 |
| AAG02357.1 | polyketide\_synthase | BGC0000963 | Bleomycin\_biosynthetic\_gene\_cluster | 29 | 33.345145287 | 336 | 2.4e-91 |
| BAE93739.1 | type\_I\_polyketide\_synthase-related\_protein | BGC0000164 | Tetronomycin\_biosynthetic\_gene\_cluster | 33 | 23.8837703756 | 336 | 2.4e-91 |
| AHB38498.1 | EpxE | BGC0000346 | Epoxomicin\_biosynthetic\_gene\_cluster | 31 | 29.5535081502 | 336 | 3.2e-91 |
| CBJ89766.1 | Polyketide\_synthase\_involved\_in\_xenocoumacin\_synthesis | BGC0001054 | Xenocoumacin\_biosynthetic\_gene\_cluster | 30 | 30.7228915663 | 334 | 9.2e-91 |
| AAS92537.1 | PKS1 | BGC0001044 | Sirodesmin\_biosynthetic\_gene\_cluster | 28 | 35.0460666194 | 334 | 9.2e-91 |
| AHV78247.1 | LasS2 | BGC0001245 | Lasiodiplodin\_biosynthetic\_gene\_cluster | 29 | 33.0262225372 | 334 | 1.2e-90 |
| CAA73127.1 | HMWP1\_protein | BGC0000467 | Yersiniabactin\_biosynthetic\_gene\_cluster | 29 | 32.7072997874 | 331 | 7.8e-90 |
| CAJ87591.1 | putative\_peptide/polyketide\_synthetase | BGC0001055 | Yersiniabactin\_biosynthetic\_gene\_cluster | 29 | 32.7072997874 | 330 | 2.3e-89 |
| CDG12864.1 | non-ribosomal\_peptide\_synthetase | BGC0001415 | Althiomycin\_biosynthetic\_gene\_cluster | 29 | 30.0141743444 | 329 | 3e-89 |
| BAD22832.1 | polyketide\_synthase | BGC0001265 | Melanin\_biosynthetic\_gene\_cluster | 28 | 33.8058114812 | 329 | 3e-89 |
| AAN59953.1 | polyketide\_synthase\_1 | BGC0001258 | 1,8-dihydroxynaphthalene\_biosynthetic\_gene\_cluster | 27 | 33.6640680369 | 329 | 3.9e-89 |
| AAS89999.1 | PksA | BGC0000007 | Aflatoxin\_biosynthetic\_gene\_cluster | 28 | 33.0970942594 | 329 | 5.1e-89 |
| CAJ76298.1 | putative\_hybrid\_polyketide-non-ribosomal\_peptide\_synthetase | BGC0000972 | Colibactin\_biosynthetic\_gene\_cluster | 30 | 29.1991495393 | 326 | 3.3e-88 |
| ABS90470.1 | NRPS/PKS | BGC0001106 | Oxazolomycin\_biosynthetic\_gene\_cluster | 36 | 21.6158752658 | 326 | 4.3e-88 |
| AAS90093.1 | PksA | BGC0000006 | Aflatoxin\_biosynthetic\_gene\_cluster | 28 | 33.0616583983 | 325 | 5.6e-88 |
| AAS90022.1 | PksA | BGC0000008 | Aflatoxin\_biosynthetic\_gene\_cluster | 27 | 33.0616583983 | 324 | 9.5e-88 |
| BAE71314.1 | polyketide\_synthase | BGC0000004 | Aflatoxin\_biosynthetic\_gene\_cluster | 27 | 33.0616583983 | 324 | 1.6e-87 |
| AAD38786.1 | polyketide\_synthase | BGC0001257 | 1,3,6,8-tetrahydroxynaphthalene\_biosynthetic\_gene\_cluster | 28 | 33.0262225372 | 323 | 2.8e-87 |
| AAS90047.1 | PksA | BGC0000009 | Aflatoxin\_biosynthetic\_gene\_cluster | 27 | 33.0616583983 | 321 | 8.1e-87 |
| AAT69682.1 | polyketide\_synthase | BGC0001247 | Cercosporin\_biosynthetic\_gene\_cluster | 27 | 33.345145287 | 318 | 8.9e-86 |
| AAP42872.1 | NanA9 | BGC0000105 | Nanchangmycin\_biosynthetic\_gene\_cluster | 33 | 23.4585400425 | 315 | 7.6e-85 |
| CDM36726.1 | Beta-ketoacyl\_synthase | BGC0001360 | Mycophenolic\_acid\_biosynthetic\_gene\_cluster | 27 | 38.9085754784 | 314 | 1.3e-84 |
| ADI59532.1 | CorJ | BGC0001091 | Corallopyronin\_biosynthetic\_gene\_cluster | 34 | 20.3756201276 | 312 | 3.8e-84 |
| ACH72912.1 | AflC | BGC0000011 | Aflatoxin/sterigmatocystin\_biosynthetic\_gene\_cluster | 28 | 32.955350815 | 312 | 6.4e-84 |
| AAY32964.1 | DszA | BGC0001093 | Disorazole\_biosynthetic\_gene\_cluster | 31 | 24.5924875974 | 310 | 1.9e-83 |
| AKQ22699.1 | malonyl\_CoA-acyl\_carrier\_protein\_transacylase | BGC0001186 | Misakinolide\_biosynthetic\_gene\_cluster | 31 | 23.5294117647 | 310 | 1.9e-83 |
| AHB38509.1 | EpnH | BGC0000345 | Eponemycin\_biosynthetic\_gene\_cluster | 28 | 32.0694542877 | 309 | 5.4e-83 |
| ABC34675.1 | polyketide\_synthase,\_putative | BGC0000186 | Thailandamide\_biosynthetic\_gene\_cluster | 34 | 21.6513111269 | 305 | 4.6e-82 |
| AFX60313.1 | polyketide\_synthase | BGC0001031 | Oocydin\_A\_biosynthetic\_gene\_cluster | 33 | 21.3678242381 | 305 | 6e-82 |
| AKA59437.1 | polyketide\_synthase | BGC0001202 | Landepoxcin\_biosynthetic\_gene\_cluster | 30 | 29.7306874557 | 305 | 6e-82 |
| AAT70103.1 | CurH | BGC0001165 | Curacin\_biosynthetic\_gene\_cluster | 31 | 29.1637136782 | 304 | 1.3e-81 |
| AEE88282.1 | CurH | BGC0000976 | Curacin\_biosynthetic\_gene\_cluster | 31 | 29.1637136782 | 304 | 1.3e-81 |
| CBJ89764.1 | Polyketide\_synthase\_involved\_in\_xenocoumacin\_synthesis | BGC0001054 | Xenocoumacin\_biosynthetic\_gene\_cluster | 28 | 32.8844790928 | 302 | 3.9e-81 |
| BAV19379.1 | polyketide\_synthase | BGC0001390 | LL-Z1272beta\_biosynthetic\_gene\_cluster | 30 | 27.6399716513 | 302 | 6.6e-81 |
| AKA59448.1 | polyketide\_synthase | BGC0001203 | Clarexpoxcin\_biosynthetic\_gene\_cluster | 31 | 29.0928419561 | 302 | 6.6e-81 |
| ABI91469.1 | beta-ketoacyl\_synthase | BGC0001094 | Enacyloxin\_biosynthetic\_gene\_cluster | 32 | 23.635719348 | 300 | 1.5e-80 |
| ACN69992.1 | polyketide\_synthase | BGC0000079 | Indanomycin\_biosynthetic\_gene\_cluster | 32 | 23.6711552091 | 299 | 4.3e-80 |
| AGN74892.1 | nonribosomal\_peptide\_synthetase/polyketide\_synthase\_hybrid\_protein | BGC0000459 | Griseoviridin\_/\_viridogrisein\_biosynthetic\_gene\_cluster | 36 | 20.2338766832 | 299 | 4.3e-80 |
| CBJ89760.1 | Polyketide\_synthase\_involved\_in\_xenocoumacin\_synthesis | BGC0001054 | Xenocoumacin\_biosynthetic\_gene\_cluster | 29 | 29.7661233168 | 295 | 4.8e-79 |
| ADY00130.1 | polyketide\_synthase | BGC0000104 | Mycophenolic\_acid\_biosynthetic\_gene\_cluster | 30 | 28.4904323175 | 294 | 1.4e-78 |
| XP\_011392701.1 | hypothetical\_protein | BGC0001281 | Ustilagic\_acid\_biosynthetic\_gene\_cluster | 25 | 35.3295535082 | 290 | 2e-77 |
| CCA89329.1 | trans-AT\_type\_I\_polyketide\_synthase | BGC0001111 | Rhizopodins\_biosynthetic\_gene\_cluster | 34 | 20.1275690999 | 289 | 3.4e-77 |
| CTQ34883.1 | AtcF;\_polyketide\_synthase,\_modules\_8-10 | BGC0001301 | Anthracimycin\_biosynthetic\_gene\_cluster | 34 | 20.5173635719 | 286 | 3.8e-76 |
| CAG23978.1 | polyketide\_synthase\_type\_I | BGC0000176 | Difficidin\_biosynthetic\_gene\_cluster | 33 | 20.0212615167 | 284 | 1.9e-75 |
| CBF80428.1 | polyketide\_synthase,\_putative\_(JCVI) | BGC0000680 | Austinol/dehydroaustinol,\_locus\_1\_biosynthetic\_gene\_cluster | 29 | 27.888022679 | 278 | 1e-73 |
| CAL69889.1 | RhiB\_protein | BGC0001112 | Rhizoxins\_biosynthetic\_gene\_cluster | 33 | 20.6236711552 | 277 | 1.3e-73 |
| AHE80994.1 | PieA4 | BGC0001169 | Piericidin\_A1\_biosynthetic\_gene\_cluster | 33 | 23.8483345145 | 276 | 3e-73 |
| AAM12909.2 | MmpA | BGC0000182 | Mupirocin\_biosynthetic\_gene\_cluster | 31 | 21.4741318214 | 272 | 4.3e-72 |
| ALA62323.1 | methylphloroacetophenone\_synthase | BGC0001309 | Usnic\_acid\_biosynthetic\_gene\_cluster | 30 | 27.7462792346 | 272 | 5.6e-72 |
| BAD08358.1 | polyketide\_synthase\_modules\_4 | BGC0000167 | Vicenistatin\_biosynthetic\_gene\_cluster | 31 | 29.8015591779 | 271 | 9.6e-72 |
| AFL48528.1 | laidlomycin\_polyketide\_synthase\_(module\_7\_and\_module\_8) | BGC0000084 | Laidlomycin\_biosynthetic\_gene\_cluster | 34 | 23.3876683203 | 269 | 4.8e-71 |
| AGN71604.1 | conidial\_yellow\_pigment\_biosynthesis\_polyketide\_synthase | BGC0000027 | Azaphilone\_biosynthetic\_gene\_cluster | 29 | 27.2147413182 | 269 | 4.8e-71 |
| EED18001.1 | polyketide\_synthase,\_putative | BGC0000154 | Stipitatic\_acid\_biosynthetic\_gene\_cluster | 29 | 29.2700212615 | 269 | 6.2e-71 |
| EAU29529.1 | hypothetical\_protein | BGC0000682 | Terretonin\_biosynthetic\_gene\_cluster | 30 | 27.2856130404 | 267 | 1.4e-70 |
| AHD05620.1 | putative\_polyketide\_synthase\_subunit | BGC0001033 | Paenilamicin\_biosynthetic\_gene\_cluster | 30 | 23.7065910702 | 265 | 6.9e-70 |
| ALI92655.1 | CitS\_citrinin\_polyketide\_synthase | BGC0001338 | Citrinin\_biosynthetic\_gene\_cluster | 26 | 33.8412473423 | 264 | 2e-69 |
| ctg1\_orf255 |  | BGC0001200 | Lorneic\_acid\_A\_biosynthetic\_gene\_cluster | 31 | 24.3798724309 | 264 | 2e-69 |
| ACN69991.1 | polyketide\_synthase | BGC0000079 | Indanomycin\_biosynthetic\_gene\_cluster | 29 | 29.7661233168 | 261 | 9.9e-69 |
| AAL01060.1 | omega-3\_polyunsaturated\_fatty\_acid\_synthase\_PfaA | BGC0000865 | Eicoseicosapentaenoic\_acid\_biosynthetic\_gene\_cluster | 26 | 34.868887314 | 260 | 1.7e-68 |
| AGY62754.1 | EbeB | BGC0000051 | Ebelactone\_biosynthetic\_gene\_cluster | 34 | 24.3444365698 | 260 | 2.2e-68 |
| ABS90471.1 | PKS\_type\_I | BGC0001106 | Oxazolomycin\_biosynthetic\_gene\_cluster | 34 | 21.8284904323 | 259 | 4.9e-68 |
| CBK62731.1 |  | BGC0001115 | Thiomarinol\_biosynthetic\_gene\_cluster | 31 | 20.0566973777 | 259 | 4.9e-68 |
| ABS90478.1 | PKS | BGC0001106 | Oxazolomycin\_biosynthetic\_gene\_cluster | 32 | 20.552799433 | 255 | 7.1e-67 |
| AGZ15474.1 | putative\_type\_I\_polyketide\_synthase | BGC0001036 | Polyoxypeptin\_biosynthetic\_gene\_cluster | 30 | 28.9510985117 | 253 | 3.5e-66 |
| AJW65408.1 | type\_I\_modular\_polyketide\_synthase | BGC0001195 | Nocardiopsin\_biosynthetic\_gene\_cluster | 29 | 28.8802267895 | 252 | 4.6e-66 |
| ADB23403.1 | polyketide\_synthase\_type\_I | BGC0001062 | TLN-05220\_biosynthetic\_gene\_cluster | 30 | 29.3054571226 | 250 | 3e-65 |
| AEZ53952.1 | polyketide\_synthase | BGC0000144 | Salinomycin\_biosynthetic\_gene\_cluster | 30 | 28.5967399008 | 249 | 5.1e-65 |
| AJW65409.1 | type\_I\_modular\_polyketide\_synthase | BGC0001195 | Nocardiopsin\_biosynthetic\_gene\_cluster | 29 | 30.4748405386 | 248 | 8.7e-65 |
| AAM12934.1 | MmpF | BGC0000182 | Mupirocin\_biosynthetic\_gene\_cluster | 31 | 23.245924876 | 247 | 1.5e-64 |
| BAH02268.1 | polyketide\_synthase | BGC0000126 | Pladienolide\_biosynthetic\_gene\_cluster | 32 | 23.9192062367 | 245 | 7.4e-64 |
| sln14 | nonribosomal\_peptide\_synthetase/polyketide\_synthase\_type\_I | BGC0001230 | Salinamides\_biosynthetic\_gene\_cluster | 29 | 23.245924876 | 243 | 2.1e-63 |
| AAO65797.1 | monensin\_polyketide\_synthase\_module\_2 | BGC0000100 | Monensin\_biosynthetic\_gene\_cluster | 32 | 22.3954642098 | 243 | 2.1e-63 |
| CCP20048.1 | divL1\_protein | BGC0001119 | Divergolide\_biosynthetic\_gene\_cluster | 30 | 24.3090007087 | 242 | 4.8e-63 |
| AGJ76601.1 | HglE | BGC0000869 | Heterocyst\_glycolipids\_biosynthetic\_gene\_cluster | 29 | 26.0807937633 | 242 | 6.2e-63 |
| BAO66519.1 | type\_I\_polyketide\_synthase | BGC0000042 | Cremimycin\_biosynthetic\_gene\_cluster | 30 | 27.9943302622 | 240 | 1.8e-62 |
| BAH02270.1 | polyketide\_synthase | BGC0000126 | Pladienolide\_biosynthetic\_gene\_cluster | 31 | 24.7696669029 | 236 | 4.5e-61 |
| BAR73007.1 | putative\_PKS\_(ACP-KS-AT-DH-KR-ACP-KS-AT-DH-ER-KR-ACP) | BGC0001194 | Hitachimycin\_biosynthetic\_gene\_cluster | 28 | 28.2423812899 | 235 | 1e-60 |
| ABP57746.1 | DepB | BGC0000993 | FK228\_biosynthetic\_gene\_cluster | 31 | 20.3756201276 | 233 | 2.2e-60 |
| ADH01485.1 | putative\_mixed\_polyketide\_synthase/non-ribosomal\_peptide\_synthetase | BGC0000995 | FR901464\_biosynthetic\_gene\_cluster | 33 | 20.4464918498 | 233 | 2.9e-60 |
| AAZ77694.1 | ChlA2 | BGC0000036 | Chlorothricin\_biosynthetic\_gene\_cluster | 30 | 24.9468462084 | 230 | 1.9e-59 |
| BAB69199.1 | modular\_polyketide\_synthase | BGC0000117 | Oligomycin\_biosynthetic\_gene\_cluster | 30 | 29.4826364281 | 228 | 9.3e-59 |
| BAF85838.1 | modular\_polyketide\_synthase | BGC0000109 | Nemadectin\_biosynthetic\_gene\_cluster | 31 | 24.6987951807 | 228 | 1.2e-58 |
| AFR69332.1 | polyketide\_synthase\_SpiB | BGC0001045 | Spiruchostatin\_biosynthetic\_gene\_cluster | 30 | 20.0212615167 | 222 | 8.7e-57 |
| ABI93779.1 | GdmPKS | BGC0000068 | Geldanamycin\_biosynthetic\_gene\_cluster | 30 | 24.415308292 | 220 | 1.9e-56 |
| AAP42873.1 | NanA11 | BGC0000105 | Nanchangmycin\_biosynthetic\_gene\_cluster | 30 | 25.3012048193 | 220 | 1.9e-56 |
| AME18003.1 | enediyne\_polyketie\_synthase | BGC0001378 | Tiancimycin\_biosynthetic\_gene\_cluster | 30 | 26.2579730687 | 215 | 6.2e-55 |
| CAJ76297.1 | putative\_polyketide\_synthase | BGC0000972 | Colibactin\_biosynthetic\_gene\_cluster | 29 | 23.4939759036 | 213 | 3.1e-54 |
| ABB86408.1 | GelA | BGC0000067 | Geldanamycin\_biosynthetic\_gene\_cluster | 31 | 22.5017717931 | 212 | 5.3e-54 |
| AAO06916.1 | GdmAI | BGC0000066 | Geldanamycin\_biosynthetic\_gene\_cluster | 31 | 22.5017717931 | 212 | 5.3e-54 |
| AAU93806.2 | polyketide\_synthase\_modules\_3\_and\_4 | BGC0000054 | Erythromycin\_biosynthetic\_gene\_cluster | 30 | 24.9822820695 | 212 | 5.3e-54 |
| BAC76492.1 | lankamycin\_synthase\_LkmAII | BGC0000085 | Lankamycin\_biosynthetic\_gene\_cluster | 29 | 23.0687455705 | 212 | 9e-54 |
| AGI61666.1 | modular\_PKS | BGC0000095 | Micromonolactam\_biosynthetic\_gene\_cluster | 28 | 20.4464918498 | 212 | 9e-54 |
| ABB88521.1 | polyketide\_synthase\_type\_I | BGC0000050 | E-837\_biosynthetic\_gene\_cluster | 30 | 22.9624379872 | 208 | 7.6e-53 |
| AAM78012.1 | warhead-forming\_iterative\_polyketide\_synthase | BGC0000112 | Neocarzinostatin\_biosynthetic\_gene\_cluster | 28 | 26.9666902906 | 206 | 4.9e-52 |
| AAY28225.1 | HbmAI | BGC0000074 | Herbimycin\_biosynthetic\_gene\_cluster | 30 | 22.466335932 | 201 | 9.3e-51 |
| CAJ76285.1 | putative\_polyketide\_synthase | BGC0000972 | Colibactin\_biosynthetic\_gene\_cluster | 27 | 23.6711552091 | 199 | 4.6e-50 |
| AAP92148.1 | EspE | BGC0000056 | Esperamicin\_biosynthetic\_gene\_cluster | 28 | 26.4351523742 | 199 | 6.1e-50 |
| AAK57185.1 | MxaB1 | BGC0001022 | Myxalamid\_biosynthetic\_gene\_cluster | 25 | 28.313253012 | 194 | 1.9e-48 |
| AAX98192.1 | polyketide\_synthase\_type\_I | BGC0000052 | ECO-02301\_biosynthetic\_gene\_cluster | 26 | 25.4429482636 | 191 | 1.6e-47 |
| AAB66505.1 | tylactone\_synthase\_module\_3 | BGC0000166 | Tylactone\_biosynthetic\_gene\_cluster | 26 | 24.8405386251 | 179 | 5e-44 |
| BAQ25512.1 | type\_I\_polyketide\_synthase | BGC0001288 | Maklamicin\_biosynthetic\_gene\_cluster | 26 | 25.584691708 | 166 | 4.3e-40 |
